# Comprehensive profiling of semi‐polar phytochemicals in whole wheat grains (*Triticum aestivum*) using liquid chromatography coupled with electrospray ionization quadrupole time‐of‐flight mass spectrometry

**DOI:** 10.1007/s11306-020-01761-4

**Published:** 2021-01-27

**Authors:** Leslie Tais, Hartwig Schulz, Christoph Böttcher

**Affiliations:** 1 Federal Research Centre for Cultivated Plants, Institute for Ecological Chemistry, Plant Analysis and Stored Product Protection, Julius Kühn-Institute, Königin-Luise-Strasse 19, 14195 Berlin, Germany; 2 Consulting & Project Management for Medicinal & Aromatic Plants, Waltraudstrasse 4, 14532 Stahnsdorf, Germany

**Keywords:** *Triticum aestivum*, Metabolomics, Tandem mass spectrometry, Hydroquinones, Hydroxycinnamic acid amides, Flavonoids, Benzoxazinoids, Lignans

## Abstract

**Introduction:**

Wheat (*Triticum aestivum*) it is one of the most important staple food crops worldwide and represents an important resource for human nutrition. Besides starch, proteins and micronutrients wheat grains accumulate a highly diverse set of phytochemicals.

**Objectives:**

This work aimed at the development and validation of an analytical workflow for comprehensive profiling of semi-polar phytochemicals in whole wheat grains.

**Method:**

Reversed-phase ultra-high performance liquid chromatography coupled with electrospray ionization quadrupole time-of-flight mass spectrometry (UHPLC/ESI-QTOFMS) was used as analytical platform. For annotation of metabolites accurate mass collision-induced dissociation mass spectra were acquired and interpreted in conjunction with literature data, database queries and analyses of reference compounds.

**Results:**

Based on reversed-phase UHPLC/ESI-QTOFMS an analytical workflow for comprehensive profiling of semi-polar phytochemicals in whole wheat grains was developed. For method development the extraction procedure and the chromatographic separation were optimized. Using whole grains of eight wheat cultivars a total of 248 metabolites were annotated and characterized by chromatographic and tandem mass spectral data. Annotated metabolites comprise hydroquinones, hydroxycinnamic acid amides, flavonoids, benzoxazinoids, lignans and other phenolics as well as numerous primary metabolites such as nucleosides, amino acids and derivatives, organic acids, saccharides and B vitamin derivatives. For method validation, recovery rates and matrix effects were determined for ten exogenous model compounds. Repeatability and linearity were assessed for 39 representative endogenous metabolites. In addition, the accuracy of relative quantification was evaluated for six exogenous model compounds.

**Conclusions:**

In conjunction with non-targeted and targeted data analysis strategies the developed analytical workflow was successfully applied to discern differences in the profiles of semi-polar phytochemicals accumulating in whole grains of eight wheat cultivars.

**Supplementary Information:**

The online version contains supplementary material available at 10.1007/s11306-020-01761-4.

## Introduction

Wheat (*Triticum* spp.) is cultivated nowadays on six continents in regions with temperate, Mediterranian-type and subtropical climates. With an annual production of 760 million tons (year 2019) it is one of the most important staple food crops worldwide and accounts for approximately 28% of the total world cereal production (http://www.fao.org/giews/, Food Outlook 2020). Common wheat (*Triticum aestivum*) and durum wheat (*Triticum turgidum* var. *durum*) are the two most cultivated wheat species, with common wheat accounting for more than 90% of the world production (Shewry 2009). About two third of the globally produced wheat are directly used for human nutrition (http://www.fao.org/giews/, Food Outlook 2020). This makes wheat a major diet component and an important source of easily digestable starch and proteins. Due to a comparatively high proportion of gluten protein, doughs made from wheat flour exhibit characteristic viscoelastic and adhesive properties that enable production of unique food products such as bread, baked goods and pasta. Because of changing dietary habits in countries undergoing industrialization and urbanization and a growing human population worldwide demand for wheat based food products is continuously rising. This is reflected by an increasing global wheat production which is estimated to reach 839 million tons in 2029 (http://www.fao.org, OECD-FAO Agricultural Outlook 2020–2029).

In addition to starch and proteins, wheat grains accumulate numerous other components which are important for human nutrition or beneficial for human health. These include nutrients (macro- and micro minerals, B vitamins), antinutrients (phytic acid), lipids, dietary fibers and a highly diverse set of phytochemicals (Zhu and Sang 2017). The latter comprise apolar compounds such as alk(en)ylresorcinols (Andersson et al. 2008), phytosterols (Nurmi et al. 2008), tocols and carotenoids (Lachman et al. 2013), semi-polar ones such as benzoxazinoids (Villagrasa et al. 2006), flavonoids (Geng et al. 2016), lignans (Smeds et al. 2007), hydroxycinnamic and hydroxybenzoic acid derivatives (Dinelli et al. 2011) as well as polar ones such as fructooligosaccharides (Verspreet et al. 2015; Nilsson et al. 1986) and amino acid betains (Corol et al. 2012; Servillo et al. 2018). Minerals, vitamins, dietary fibers and most of the phytochemicals are known to be enriched or solely located in the bran or in the germ (Liu 2007). In contrast to refined white flour almost exclusively consisting of starchy endosperm, both compartments are components of whole grain flour. There is mounting evidence that consumption of whole grain products rich in dietary fibers and phytochemicals is associated with a lower incidence of chronic diseases such as cardio-vascular disease and type 2 diabetes (Shewry and Hey 2015; Zhu and Sang 2017).

Because of the importance of wheat for human nutrition the metabolite composition of the wheat grain and its compartments is well studied. In particular for nutritionally important compound classes, a multitude of classical analytical methods have been developed in the past. Also metabolite profiling approaches in combination with targeted and non-targeted data analysis strategies have been applied to study wheat grain composition in dependence on genetic and environmental factors on metabolome level. For this purpose techniques based on ^1^H NMR spectroscopy (Shewry et al. 2017, 2018) and GC/EI-MS (Bonte et al. 2014; Francki et al. 2016; Zhen et al. 2016; Zorb et al. 2006) were applied targeting polar metabolites including free amino acids, organic acids and sugars. For profiling of apolar wheat grain metabolites such as free fatty acids, oxylipins, glycerolipids, phospholipids, glycolipids, sphingolipids, alk(en)ylresorcinols and phytosterol conjugates direct infusion ESI-QqQ-MS (Gonzalez-Thuillier et al. 2015) and UHPLC/API-high resolution accurate mass tandem-MS (Geng et al. 2015; Riewe et al. 2017) were used as analytical platforms. The latter is also well suited for profiling of semi-polar wheat grain metabolites as it facilitates sensitive detection of numerous compound classes (Koistinen and Hanhineva 2017) and provides capabilities for dereplication of already known and for structural characterization of yet unknown analytes. However, profiling approaches for semi-polar metabolites described in the literature so far are often restricted to individual compound classes such as phenolics (Dinelli et al. 2011), flavonoids (Geng et al. 2016) or benzoxazinoids (Hanhineva et al. 2011).

In order to fill this gap an analytical workflow for comprehensive profiling of semi-polar phytochemicals from whole wheat grains on basis of UHPLC/ESI-QTOFMS was developed. The workflow was validated using a set of representative model compounds and exemplarily applied to compare profiles of semi-polar phytochemicals of eight wheat cultivars using non-targeted and targeted data analysis approaches. In addition, a comprehensive collection of chromatographic and mass spectral data for 248 wheat grain metabolites was assembled in the course of this study.

## Materials and methods

### Plant material and chemicals

Wheat grains were obtained from a field trial conducted at the experimental station of Strube Research GmbH & Co. KG located in Söllingen, Germany (altitude 105 m a.s.l.; mean annual temperature 9.7 °C; mean annual precipitation 626 mm; black earth/loess). Eight soft winter wheat cultivars (*Triticum aestivum* L. ‘Appertus’, ‘Attraktion’, ‘Capone’, ‘Dichter’, ‘JB Asano’, ‘Julius’, ‘Patras’, ‘Spontan’) were grown on a total of 32 plots (8 cultivars × 4 agronomic replicates; plot size, 10 m^2^) which were arranged according to a randomized block design with four blocks. Seeds were sown on October 11th, 2015 at a density of 350 seeds m^− 2^. Fertilization (200 kg N ha^− 1^ applied as calcium ammonium nitrate or ammonium sulfate) and plant protection were performed according to good agricultural practice. Grains were harvested on August 8th, 2016.

Ethanol (≥ 99.9%, for HPLC), methanol (≥ 99.95%, for LC-MS) and acetonitrile (≥ 99.95%, for LC-MS) were purchased from Th.Geyer (CHEMSOLUTE, http://www.thgeyer.com). Ultra-pure water (resistivity ≥ 18.2 MΩ cm) was obtained from a water purification system (Arium 611, http://www.sartorius.de). Formic acid (≥ 98%, for LC-MS), ammonium formate (≥ 99%, for MS) and ammonium acetate (≥ 99%, for LC-MS) were supplied by Sigma-Aldrich (http://www.sigmaaldrich.com), acetic acid (≥99.5%, p.a.) by Th.Geyer. Reference compounds used for authentication of metabolites, as model compounds for method validation or internal standards are listed in Supplemental Table 1.

### Sample preparation

Wheat grains (250 g) were homogenized using a cross beater mill (Retsch SK1, www.retsch.com) equipped with a 200 µm sieve. The resulting fine powder was thoroughly mixed by manual stirring. Afterwards an aliquot (50 g) was dried to constant weight *in vacuo* at ambient temperature for 3 days using a freeze dryer (Christ Gamma 1–16 LSC, condenser temperature − 50 °C, pressure 0.04 mbar, http://www.martinchrist.de). The dried flour was stored in sealed 100-mL polyethylene wide mouth bottles in a freezer at − 80 °C until extraction.

### Extraction

Dried wheat flour was thoroughly mixed with a spatula and an aliquot of (500 ± 10) mg was weighed into a 15-mL polypropylene centrifuge tube. After addition of 500 µL of the internal standard mix (50 µM 4-aminohippuric acid, 5 µM kinetin, 25 µM aspartame, 10 µM biochanin A in ethanol/water, 1/1 (v/v)) and 10 mL ethanol/water, 4/1 (v/v) the mixture was briefly vortexed, sonicated (15 min, 22–25 °C, Sonorex RK106, http://bandelin.com) and shaken (100 min, 1800 min^− 1^, 22 °C). Following centrifugation (10 min, 4696 × *g*, 22 °C) the resulting supernatant was transferred into a 25-mL volumetric flask. The remaining residue was resuspended in 10 mL ethanol/water, 4/1 (v/v) and extracted a second time following the above described procedure. Both supernatants were combined and their volume adjusted to 25 mL using ethanol/water, 4/1 (v/v). After thorough mixing 1500 µL of the resulting stock extract were transferred into a 2-mL polypropylene tube and evaporated to dryness using a centrifugal vacuum concentrator (40 °C, 4 mbar, Christ RVC 2–18). Two-hundred µL methanol/water, 4/1 (v/v) were added to the remaining residue. The mixture was shaken (10 min, 1800 min^− 1^, 22 °C), sonicated (10 min, 22 °C) and incubated in a fridge at 6 °C to precipitate starch and proteins. After 16–18 h, the mixture was centrifuged (10 min, 13,000×*g*, 6 °C) and the supernatant transferred into a vial, which was stored in a fridge at 6 °C until analysis.

### UHPLC/ESI-QTOFMS

LC/MS analyses were performed on an Infinity 1290 series UHPLC system (Agilent Technologies, http://www.agilent.com) consisting of a binary pump (G4220A), an autosampler (G4226A, 20 µL loop), an autosampler thermostat (G1330B) and a thermostatted column compartment (G1316C) which was interfaced to an iFunnel Q-TOF mass spectrometer (G6550A, Agilent Technologies) via a dual Agilent jet stream electrospray ion source. MassHunter LC/MS Data Acquisition software was used for controlling the instrument and data acquisition, MassHunter Qualitative and Quantitative Analysis software for data evaluation. The mass spectrometer was operated in low mass range (*m/z* 1700) and extended dynamic range (2 GHz) mode. The slicer mode was set to high sensitivity. Using these settings, the mass resolution (full width at half maximum) at *m/z* 922 was approximately 23,000. The instrument was auto tuned and calibrated according to manufacturer’s recommendations using ESI-L tuning mix (Agilent Technologies).

Extracts (1 µL injection volume) were separated on a Zorbax RRHD Eclipse Plus C18 column (100 × 2.1 mm, 1.8 µm particle size, Agilent Technologies) using 0.1% (v/v) formic acid in water and 0.1% (v/v) formic acid in methanol as eluent A and B, respectively. The following binary gradient program at a flow rate of 400 µL min^− 1^ was applied: 0–15 min, linear from 5 to 65% B; 15–16 min, linear from 65–100% B; 16–18 min, isocratic, 100% B; 18–20 min, isocratic, 5% B. The column temperature was maintained at 40 °C and the autosampler temperature at 6 °C. Mass spectra were acquired from *m/z* 80-1700 in centroid mode using an acquisition rate of 2 spectra per second. The following instrument settings were applied for positive (negative) ion mode: nebulizer gas, nitrogen, 35 psig; dry gas, nitrogen, 200 °C, 18 L min^− 1^; sheath gas, nitrogen, 300 °C, 12 L min^− 1^; capillary voltage − 3000 V (3000 V); nozzle voltage 0 V (0 V); high pressure funnel, voltage drop 200 V, RF voltage 150 V; low pressure funnel, voltage drop 100 V, RF voltage 100 V; funnel exit DC 50 V; octopole RF voltage 750 V; collision gas, nitrogen; collision energy 0 V.

Collision-induced dissociation (CID) mass spectra were acquired in targeted-MS/MS mode using scheduled precursor ion lists and the following parameters: acquisition rate MS, 3 spectra per second; acquisition rate MS/MS, 3 spectra per second, isolation width, narrow (1.3 *m/z*); collision energy, 10–60 V; collision gas, nitrogen. For acquisition of CID mass spectra of in-source fragment ions (pseudo-MS^3^) funnel exit DC voltage was increased to 120 V.

Reference mass correction was used for accurate mass measurements and tandem-MS experiments. For this purpose, a solution of purine (20 µM) and hexakis-(2,2,3,3-tetrafluoropropoxy)phosphazine (20 µM) in acetonitrile/water, 95/5 (v/v) was continuously introduced through the second sprayer of the dual ion source at a flow rate of 20 µL min^− 1^ using an external HPLC pump (PU-980, JASCO) equipped with a 1:100 splitting device.

### LC/MS data acquisition

LC/MS data acquisition was carried out in analytical batches with a maximum of 95 samples (∼33 h of instrument time). To correct for systematic instrumental drift within and between analytical batches a quality control (QC) sample approach was applied. For this purpose, a pooled QC sample was prepared by mixing aliquots of all biological samples to be analyzed within the frame of an experiment. Analytical batches were structured as follows: seven QC samples at the beginning, two QC samples at the end and in-between segments of four randomly selected samples which were bracketed by two QC samples. Instrument maintenance (cleaning of the ion source and the LC column, instrument tuning, mass calibration) was performed at the beginning of each analytical batch. In case multiple analytical batches have to be analyzed consecutively in the frame of an experiment, maintenance operations between analytical batches were restricted to LC column cleaning and mass calibration.

### Instrumental drift correction and normalization

Instrumental drift correction and normalization was carried out individually for each molecular feature (*m/z*-retention time pair) and each analytical batch. For this purpose, a correction curve was constructed using the intensity of the molecular feature in QC samples, their injection order and a LOWESS/Spline interpolation algorithm (Tsugawa et al. 2014; Broadhurst et al. 2018). Afterwards the intensity of the molecular feature in QC and biological samples was normalized to this correction curve. The smoothing parameter ‘Span’ was individually optimized for each analytical batch using the implemented optimization procedure. The first five QC samples injected at the beginning of each analytical batch for equilibration of the analytical platform were not considered for drift correction.

### Method validation

#### Recovery rate and matrix effect

Recovery rate and matrix effect were evaluated for ten model compounds at two different concentrations. To determine appropriate spiking concentrations, the linear range of each model compound was determined in positive and negative ion mode by analysis of dilution series prepared in methanol/water, 1/1 (v/v) (Supplemental Table 2). Based on these data the following mixture was prepared in ethanol/water, 4/1 (v/v): 6.7 µM 4-aminohippuric acid, 66.7 µM 3-indoxyl-β-d-glucoside, 1.7 µM kinetin, 3.3 µM aspartame, 1.7 µM umbelliferone, 16.7 µM indole-3-carboxylic acid, 3.3 µM vitexin, 6.7 µM rutin, 33.3 µM 4-methoxycinnamic acid, 3.3 µM biochanin A. This mixture was used to spike wheat samples before (A, n = 4) and after extraction (B, n = 4) at a high (1000 µL) and a low (100 µL) concentration. In addition, spiked solvent blanks (C, n = 4 for each concentration level) and non-spiked wheat samples (D, n = 4) were prepared. All samples were analyzed in two analytical batches by UHPLC/ESI-QTOFMS in positive and negative ion mode as described in paragraph 2.5. Model compounds were quantified by integration of extracted ion chromatograms generated for respective quantifier ions (Supplemental Table 2). Afterwards, QC sample-based instrumental drift correction and normalization was performed. Recovery rates were expressed for each model compound and concentration as (average drift-corrected peak area of samples A − average drift-corrected peak area of samples D) / (average drift-corrected peak area of samples B – average drift-corrected peak area of samples D). Matrix effects were expressed as (average drift-corrected peak area of samples B – average drift-corrected peak area of samples D) / (average drift-corrected peak area of samples C).

#### Repeatability

To assess intra- and inter-batch repeatability a whole wheat grain homogenate (‘JB Asano’) was repeatedly analyzed in five batches within a period of two weeks. In each batch five extracts were prepared and analyzed in quadruplicate as described in paragraph 2.5 by UHPLC/ESI-QTOFMS in positive and negative ion mode. Full instrument maintenance (cleaning of the ion source and the LC column, instrument tuning, mass calibration) was carried out at the beginning of each analytical batch. As QC sample aliquots of extracts prepared in the first batch were pooled. Aliquots of the resulting QC sample were stored in a fridge at 6 °C and used across the entire experiment. Test compounds were quantified by integration of extracted ion chromatograms generated for respective quantifier ions (Supplemental Table 3). After normalization by sample dry weight peak areas were corrected for instrumental drift and normalized for each analytical batch as described in paragraph 2.6. Variance levels were estimated as detailed in Supplemental Table 4 using a nested linear random-effects model.

#### Linearity

For evaluation of the linear range wheat grain extracts with different concentrations (11 levels, 4–400 µg dry weight per µL extract) were prepared in duplicate and analyzed by UHPLC/ESI-QTOFMS in positive and negative ion mode. Forty-three test metabolites were quantified by integration of extracted ion chromatograms generated for respective quantifier ions (Supplemental Table 5). Afterwards, the linear range was estimated by visual inspection of the resulting calibration curves. Using a linear calibration model (y = ax), the coefficient of determination was determined within the estimated linear range using MassHunter Quantitative Analysis software.

#### Accuracy

Aliquots of a whole wheat grain extract (‘JB Asano’) were spiked with six model compounds at ten different concentration levels (100%, 105%, 110%, 115%, 120%, 125%, 150%, 200%, 400% and 800%) in duplicate. With respect to the linear ranges of the model compounds following concentrations were used for the lowest level (100%): 0.5 µM 4-aminohippuric acid, 2 µM 3-indoxyl-β-d-glucoside, 0.1 µM kinetin, 0.2 µM aspartame, 0.2 µM umbelliferone, 0.5 µM biochanin A. Spiked wheat grain extracts were analyzed by UHPLC/ESI-QTOFMS in positive ion mode in three analytical batches over a period of one week as described in paragraph 2.5. Model compounds were quantified by integration of extracted ion chromatograms generated for respective quantifier ions (Supplemental Table 2). Peak areas were corrected for instrumental drift and normalized as described in paragraph 2.6. Drift-corrected peak areas of the two technical replicates analyzed in one batch were averaged. Afterwards, the averages thus obtained from three analytical batches were used for calculation of fold-changes and *P* values (Supplemental Table 6). Fold-changes between two concentration levels were calculated as ratio of the means. To evaluate whether the means of two concentration levels are significantly different Student’s *t*-test (two-tailed, equal variances) was applied.

### Metabolite profiling

#### Data acquisition

Homogenized and dried wheat grain samples were extracted in duplicate. The resulting 64 extracts (8 cultivars × 4 agronomic replicates × 2 technical replicates) and two blank samples were analyzed as described in paragraph 2.5 by UHPLC/ESI-QTOFMS in positive and negative ion mode in one analytical batch, each. To check the quality of the obtained raw data retention times and abundances of spiked internal standards were evaluated.

#### Non‐targeted data analysis

Raw data files were converted into *mzData* format using MassHunter Qualitative Analysis software, arranged in nine sample classes (8 cultivars + QC) and processed using the R package *XCMS *(Smith et al. 2006). Feature detection was performed using the *centWave* algorithm (Tautenhahn et al. 2008) [parameters: prefilter = (3, 1000); sntresh = 3; ppm = 25; peak width = (5, 12)]. Alignment was accomplished by consecutive application of the functions *group.density* (parameters: minfrac = 1; bw = 2; mzwid = 0.02), *retcor.loess* (parameters: span = 1; missing = 0; extra = 0) and *group.density* (parameters: minfrac = 0.75; bw = 1, mzwid = 0.02). Missing feature intensities were estimated using the function *fillPeaks*. Features with low repeatability in pooled QC samples (CV of feature intensities > 30%) were excluded from further analysis. Feature intensities were normalized by sample weight, corrected for instrumental drift and normalized as described in paragraph 2.6. Principal component analyses were performed on log_2_-transformed and mean centered data without further scaling using the function *pca* (parameters: nPcs = 5; method = ‘svd’; scale = ‘none’; center = TRUE) from the R package *pcaMethods *(Stacklies et al. 2007). To evaluate the effect of the cultivar on the feature intensity sample weight normalized and instrumental drift-corrected feature intensities of the two technical replicates were averaged and log_2_-transformed. A one-factorial fixed effect model was fitted to the transformed feature intensities using the function *lm* from the R package *stats* and analyzed by the function *anova* from the same package. The resulting *P* values were corrected for multiple testing using the function *p.adjust* (method = ‘fdr’) from the package *stats*.

#### Targeted analysis

Target metabolites were quantified by integration of extracted ion chromatograms generated for respective quantifier ions (Supplemental Tables 7a–j). Target compounds with low intensity (mean intensity < 2000) and low repeatability (CV of intensities > 30%) in QC samples were excluded from further analysis. Feature intensities were normalized by sample weight, corrected for instrumental drift and normalized according to paragraph 2.6 and log_2_-transformed. Principal component analysis was performed as described above. For hierarchical cluster analyses (Euclidean distance, average linkage method) and generation of heatmaps sample weight normalized and drift-corrected feature intensities of technical and agronomic replicates were averaged, log_2_-transformed and subjected to MultiExperiment Viewer 4.9 (Saeed et al. 2003).

## Results and discussion

### Optimization of the extraction procedure

The bulk of a wheat grain is composed of starch and gluten proteins. These storage proteins can be classified according to their solubility into glutenins and gliadins. Since gliadins are soluble in aqueous alcohols (Urade et al. 2018) which represent typical solvent mixtures used in plant metabolomics to extract polar and semi-polar metabolites the extraction procedure requires optimization in order to maximize the yield of semi-polar metabolites while minimizing the amount of co-extracted matrix components. To this end a whole wheat grain homogenate was extracted with nine different mixtures of water, organic solvent (methanol, ethanol and acetonitrile) and formic acid at a solid-to-solvent ratio of 1/20 (w/v). The extracts obtained were evaporated to dryness and the resulting non-volatile residues (NVRs) were gravimetrically quantified (Supplemental Fig. 1). Afterwards, NVRs were redissolved in methanol/water, 4/1 (v/v) and analyzed by LC/MS to assess distribution and abundance of semi-polar metabolites extracted.

At a solvent-to-water ratio of 4/1 (v/v) the aprotic solvent acetonitrile gave significantly lower quantities of NVRs compared to the protic solvents methanol and ethanol. Decrease of the solvent-to-water ratio from 4/1 to 1/1 (v/v) led in case of ethanol and acetonitrile to massively increased quantities of NVRs. Likewise, addition of 0.1% (v/v) formic acid at a solvent-to-water ratio of 4/1 (v/v) resulted in drastically increased amounts of NVRs for both alcohols, but not for acetonitrile.

Visual inspection of deconvoluted total compound chromatograms did not reveal obvious qualitative differences between extracts prepared with different solvent mixtures. Nevertheless, quantitative differences were found for acylated biogenic amines such as monoacylated putrescine (HCAA2) and agmatine (HCAA11) conjugates as well as diacylated spermine conjugates (HCAA20, Supplemental Fig. 2a, Supplemental Table 7b). Alcohol-water mixtures at a ratio of 1/1 (v/v) exhibited low extraction efficiencies for these basic metabolites. Use of alcohol-water mixtures at a ratio of 4/1 (v/v) resulted in significantly higher extraction efficiencies which could be further increased by acidification with 0.1% (v/v) formic acid. In comparison to the tested alcohol-water mixtures, acetonitrile-water mixtures displayed a comparatively poorer performance for extraction of biogenic amine conjugates.

Further differences in the extraction efficiency of different solvent mixtures were observed for glycosylated benzoxazinoids and hydroquinones. In extracts prepared with alcohol-water mixtures at a ratio of 4/1 (v/v) the level of 2,4-dihydroxy-1,4-benzoxazin-3-one (DIBOA) hexoside BX3 was near the detection limit (Supplemental Fig. 2b, Supplemental Table 7c). When using alcohol-water mixtures at a ratio of 1/1 (v/v) or acetonitrile-water mixtures for extraction substantial amounts of BX3 became detectable and coincided with reduced levels of DIBOA dihexoside BX4. A similar negative correlation was found for di- (HQ4) and triglycoslyated methoxyhydroquinone (HQ2) (Supplemental Fig. 2c, Supplemental Table 7a). To test whether this degradation was attributed to enzymatic activities extracts were prepared with acetonitrile/water, 4/1 (v/v) at 22 °C and 75 °C and levels of glycosylated benzoxazinoids and hydroquinones quantified (Supplemental Fig. 2d). Formation of monoglycosylated BX3 and diglycoslyated HQ4 was strongly suppressed upon thermal denaturation by increase of the extraction temperature, which suggests that enzymatic degradation occured during the early stage of extraction at room temperature until quenching by organic solvent.

Of the nine tested solvent mixtures ethanol/water, 4/1 (v/v) was decided to be used for extraction of semi-polar metabolites from whole wheat grain homogenate. This solvent mixture resulted in intermediate quantities of co-extracted matrix (NVR 82 mg/g dry weight, range of all tested solvent mixtures 45–131 mg/g dry weight) while exhibiting good extraction efficiencies for basic acylated biogenic amines and good quenching capabilities to suppresses enzymatic deglycosylation during extraction at ambient temperature.

Using the optimized solvent mixture the extraction efficiency dependent on the number of extraction steps was investigated. Therefore whole wheat grain homogenate was consecutively extracted three times with ethanol/water, 4/1 (v/v) at a solid-to-solvent ratio of 1/20 (w/v). The resulting extracts were analyzed by LC/MS and eight abundant semi-polar metabolites exemplarily quantified (Supplemental Fig. 3). The first and the second extraction step yielded on average 86% and 12% of the total amount obtained in three consecutive extraction steps, respectively. This suggested that an almost quantitative recovery of the tested semi-polar metabolites was achieved with two consecutive extraction steps.

Due to the comparably low amount of semi-polar metabolites present in whole wheat grains and the low solid-to-solvent ratio used for extraction, the concentration of semi-polar metabolites in the primary extract obtained after two extraction steps was not sufficient for comprehensive LC/MS profiling. In addition, ethanol/water, 4/1 (v/v) is characterized by a high elution strength in reversed-phase chromatography which leads to low resolution in the front area of the chromatogram when used as injection solvent. Therefore, concentration of the primary extract (factor 7.5) together with a change of the solvent was required to generate a sample compatible with reversed-phase LC/MS profiling. When using methanol/water, 4/1 (v/v) for solubilization of the non-volatile residue of the primary extract it was found, that co-extracted starch and proteins could be precipitated upon storage in a fridge overnight, which substantially reduced the matrix load of the final extract. A stepwise scheme of the optimized extraction procedure is given in Supplemental Fig. 4.

### Optimization of the chromatographic separation

To optimize the chromatographic separation of the semi-polar metabolite fraction on a conventional C18 reversed phase (Zorbax Eclipse Plus, 2.1 mm × 100 mm, 1.8 µm particle size) the effect of different organic modifiers (methanol, acetonitrile) and gradient programs (steepness 4, 5, 6% eluent B min^− 1^) on the distribution of separated metabolites was evaluated in a first step. A predefined chromatographic cycle time of 20 minutes was used to enable later medium-throughput metabolite profiling. Due to its lower elution strength in comparison to acetonitrile, methanol facilitated a stronger dispersion of the chromatographic region of semi-polar metabolites and a slightly better retention of polar ones (Supplemental Fig. 5). Using a gradient with a starting point of 5% methanol and a slope of 4% methanol min^− 1^ chromatographic regions containing gluten proteins and polar lipids were almost completely excluded from MS data acquisition.

In a second step different eluent additives (formic acid, ammonium formate, acetic acid and ammonium acetate) were evaluated using the optimized methanol-water gradient. Within the tested pH range of 2.9 (0.1% (v/v) formic acid) to 6.8 (10 mM ammonium acetate) the influence of the eluent pH on retention behavior and chromatographic peak shape of neutral metabolites such as glycosylated hydroquinones, benzoxazinoids, flavones and flavonols was low. In contrast, strong effects were observed for basic metabolites such as monoacylated putrescine, spermidine and agmatine conjugates as well as diacylated spermine conjugates (Supplemental Fig. 6a, b). For these compounds, increase of the eluent pH resulted in increased retention, peak tailing and reduced sensitivity upon electrospray ionization in positive ion mode. However, due to the presence of hydrophobic hydroxycinnamoyl moieties retention of acylated biogenic amines on the applied reversed phase was sufficient when using 0.1% (v/v) formic acid (pH 2.9) or 0.1% (v/v) acetic acid (pH 3.3) as eluent additives. A strong pH dependence of the retention behavior was also observed for hydroxybenzoic/-cinnamic acids and α,ω-dicarboxylic acids (Supplemental Fig. 5c, d ). As weak acids (pK_a_ 4.2–4.6) they were almost completely dissociated at pH > 6 and therefore exhibited poor retention on the applied reversed phase when using 10 mM ammonium formate (pH 6.2) or ammonium acetate (pH 6.8) as eluent additives. In contrast, application of 0.1% (v/v) formic acid or 0.1% (v/v) acetic acid as eluent additives suppressed dissociation and substantially increased retention of these metabolites. Due to the retention behavior and symmetric peak shape of basic acylated biogenic amines and weak acids at pH ≈ 3, 0.1% (v/v) formic acid and 0.1% (v/v) acetic acid proved to be the best among the tested eluent additives. Comparison of the electrospray efficiencies of selected neutral, basic and acidic metabolites in positive and negative ion mode did not reveal clear differences between both acids. Formic acid was finally chosen since it is routinely applied in our laboratory as eluent additive in reversed-phase LC/MS.

### Annotation of semi‐polar metabolites

**Fig. 1:**
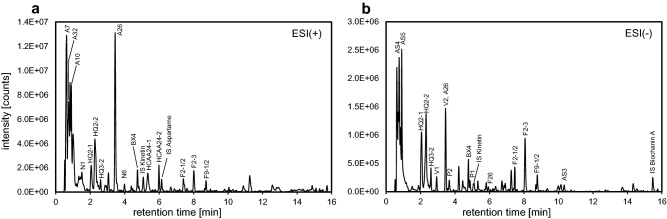
Representative total compound chromatograms obtained from a hydroethanolic whole wheat grain extract (‘JB Asano’) using UHPLC/ESI-QTOFMS in positive (**a**) and negative (**b**) ion mode. Total compound chromatograms were reconstructed from raw data using the algorithm “Find Compounds by Molecular Feature“ implemented in MassHunter Qualitative Data Analysis. For compound labelling see Supplemental Table 7. IS, internal standard

In order to comprehensively characterize the semi-polar metabolite fraction whole grains of eight soft winter wheat cultivars were extracted using the established protocol and analyzed together with blank extracts by UHPLC/ESI-QTOFMS in positive and negative ion mode. Representative chromatograms are shown in Fig. 1. The resulting raw data files were subjected to feature detection and alignment using the XCMS package (Smith et al. 2006). Subsequently, compound spectra were deconvoluted and adduct ions annotated by means of the CAMERA package (Kuhl et al. 2012). Mass-to-charge ratios and retention times of features which were annotated as protonated/deprotonated molecular ion and had sufficient intensity and did not occur in blank extracts were arranged in scheduled precursor lists. These lists were used in targeted tandem-MS experiments for acquisition of collision-induced dissociation (CID) mass spectra at multiple collision energies.

Based on accurate mass and isotope pattern putative elemental compositions were calculated for each pseudomolecular ion included in the scheduled precursor ion list and used to query compound databases such as ChemSpider, PubChem and KEGG. In a next step, the obtained accurate mass CID mass spectra were matched against reference spectra collected in spectral libraries such as METLIN (Guijas et al. 2018) or MassBank (Horai et al. 2010). In case of no match, CID mass spectra were manually interpreted in order to refine or confirm initial hits from compound database or literature search. To unequivocally confirm putative annotations, chromatographic and mass spectral properties of reference compounds and annotated metabolites were compared.

**Fig. 2:**
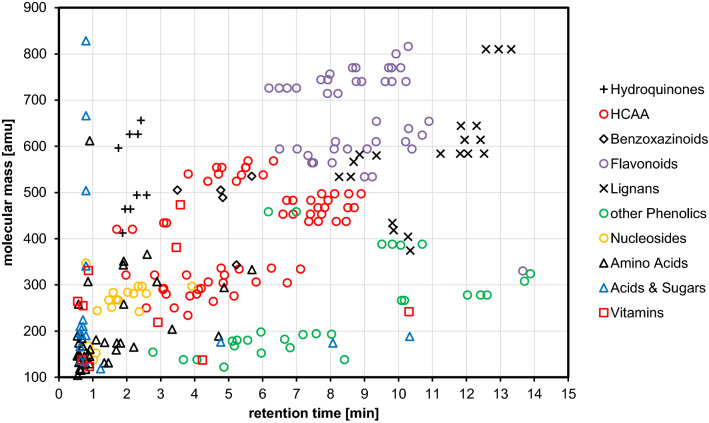
Distribution of retention times and molecular masses of 248 annotated metabolites detected in hydroethanolic whole grain extracts of eight soft winter wheat cultivars

Following the workflow described above, a total of 248 unique compounds were annotated. The distribution of their retention times and molecular masses, sorted by compound class is shown in Fig. 2. Among the annotated compounds, 56 were identified using a commercially available reference compounds (annotation level 1). By interpretation of chromatographic and mass spectral data putative structural annotations were derived for 155 compounds (annotation level 2), whereas for the remaining 37 the compound class was putatively characterized (annotation level 3). In the following sections, the structural diversity within the annotated compound classes is briefly discussed.

#### Hydroquinones

According to the literature (Zhokhov et al. 2009, 2010) a hydroquinone trihexoside (HQ1) together with di- and trihexosylated methoxyhydroquinones (HQ4, HQ2) and 2,6-dimethoxyhydroquinones (HQ5, HQ3) were annotated (see Supplemental Table 7a for analytical data). For HQ2, HQ4 and HQ5 two isomers were chromatographically separated which possibly differ in the glycosylation site of the aglycone. As discussed above, diglycosylated hydroquinones can be formed from respective triglycosylated species by enzymatic deglycosylation during sample preparation. Therefore, it is unclear whether HQ4 and HQ5 represent native metabolites or artificially formed compounds. However, since minor amounts of HQ4 and HQ5 were detected after solvent extraction at 75 °C it can be assumed that diglycosylated hydroquinones co-occur as biosynthetic intermediates in addition to the quantitatively dominating triglycoslyated species in wheat grains. Due to their high polarity glycosylated hydroquinones eluted at low retention times (t_R_ 1.7–2.6 min). They were electrospray ionized in both ion modes predominantly forming [M+NH_4_]^+^/[M+Na]^+^ or [M−H]^−^ ions, respectively. Upon CID of [M+NH_4_]^+^ ions informative fragment ion spectra were obtained. For HQ2-HQ5, protonated aglycone ions were generated by in-source fragmentation and used as precursor ions in pseudo-MS^3^ experiments. The obtained spectra were referenced against CID mass spectra obtained from [M+H]^+^ ions of commercially available methoxyhydroquinone and 2,6-dimethoxyhydroquinone. In addition to di- and trihexosylated hydroquinones, a monohexoslyated 2,6-dimethoxyhydroquinone carrying a sulfate moiety on the glycone (HQ6) was detected and mass spectrometrically characterized.

#### Hydroxycinnamic acid amides

A total of 60 metabolites were annotated as hydroxycinnamic acid-conjugated biogenic amines (see Supplemental Table 7b for analytical data). These comprise monoacylated conjugates of putrescine, hydroxyputrescine (HCAA1–4), agmatine, hydroxyagmatine, oxoagmatine (HCAA5-12) and spermidine (HCAA 13) as well as diacylated conjugates of spermidine (HCAA14–18) and spermine (HCAA19–26). As acyl moieties acetyl, coumaroyl, caffeoyl, feruloyl, 3,4-dimethoxycinnamoyl and sinapoyl were found. The chemical complexity within this compound class was further increased by the presence of geometrical and/or positional isomers. Thus, up to four isomers were chromatographically separated for diacylated spermidine conjugates. Due to the different number of acyl moieties and different amine backbones the annotated hydroxycinnamic acid amides displayed a large variation in retention time (1.7–8.9 min). As amines or guanidines they were preferentially electrospray ionized in positive ion mode forming abundant [M+H]^+^ ions, which yielded under CID conditions readily interpretable fragment ion spectra. The presence of specific hydroxycinnamoyl moieties could be often deduced from the occurrence of characteristic acylium fragment ions which resulted from cleavage of amide bonds.

#### Benzoxazinoids

In accordance with the literature (Hanhineva et al. 2011), hexosides of differently substituted 1,4-benzoxazin-3-ones (BOAs) were detected in whole wheat grain extracts (see Supplemental Table 7c for analytical data). Among them, two dihexosylated lactam-type benzoxazinoids (2-Hydroxy-BOA (BX1), 2,7-dihydroxy-BOA (BX2)) and two dihexosylated hydroxamic acid-type benzoxazinoids (2,4-dihydroxy-BOA (BX3), 2,4-dihydroxy-7-methoxy-BOA (BX5)) were annotated with BX3 being the quantitatively dominating one. Beside these dihexosides, low amounts of a monohexoside of 2,4-dihydroxy-BOA (BX4) were found, which could be formed during extraction by enzymatic deglycosation of BX3. The annotated benzoxazinoids were preferentially analyzed under negative ion electrospray conditions under which they predominantly formed [M−H]^−^ ions. In combination with published fragmentation pathways (de Bruijn et al. 2016), CID mass spectra obtained from [M−H]^−^ ions were used for structural assignments. Under positive ion electrospray conditions a more complex ion formation was observed complicating quantification. Here, [M+Na]^+^ and in-source fragment ions (of type [M+H-C_12_H_22_O_11_]^+^ for dihexosides) were predominately formed and accompanied by low abundant [M+NH_4_]^+^ and [M+H]^+^ ions.

#### Flavonoids

A total of 41 metabolites were annotated as flavonoids indicating a high structural diversity of this compound class in wheat grains (see Supplemental Table 7d for analytical data). Regarding aglycone structure and glycosylation pattern four classes of flavonoids can be distinguished: flavone di-*C*-glycosides (F1–F5), *O*-glycosylated/*O*-acylated flavone di-*C*-glycosides (F6–F11), flavone di/tri-*O*-glycosides (F12–F15, F17–F19) and flavonol di/tri-*O*-glycosides (F20–F24). In accordance with the literature (Geng et al. 2016) apigenin, luteolin and chrysoeriol were identified as aglycones in the 11 annotated flavone di-*C*-glycosides. These flavones are glycosylated at position *C*-6 and *C*-8 (Brazier-Hicks et al. 2009) either with two pentoses, two hexoses or one hexose and one pentose. Due to the presence of two glycosylation sites and incorporation of different pentoses and hexoses numerous isomers can be formed. In case of the quantitatively dominating apigenin *C*-pentoside *C*-hexosides (F2) three isomers were chromatographically separated. Another 17 conjugated flavone di-*C*-glycosides were detected which result from modification of isomeric apigenin *C*-pentoside *C*-hexosides either by *O*-hexosylation or by esterification with hydroxycinnamic acids (ferulic, 5-hydroxyferulic and sinapic acid) or hydroxybenzoic acids (vanillic and syringic acid). In addition, 7 exclusively *O*-glycosylated flavones and 5 *O*-glycosylated flavonols were detected. Flavone *O*-glycosides were derived from apigenin, luteolin, chrysoeriol and tricin, which was also found in unconjugated form (F16). Kaempferol, quercetin, isorhamnetin and syringetin were identified as aglycones in flavonol *O*-glycosides. Glycones of flavones and flavonol *O*-glycosides are composed of hexoses and deoxyhexoses. The annotated flavonoids were electrospray ionized in both ion modes mainly forming [M+H]^+^/[M+Na]^+^ or [M−H]^−^ ions, respectively. In case of *O*-glycosylated flavones and flavonols formation of in-source fragment ions was observed in positive ion mode. Upon CID of [M+H]^+^ or [M−H]^−^ ions, informative fragment ion spectra were obtained which allowed discrimination of *O*- and *C*-glycosides (Cuyckens and Claeys 2004). Due to their different stability *O*-glycosidic bonds were cleaved upon CID whereas *C*-glycosidic bonds remained intact. Characteristic fragment ions were formed in *C*-glycosides by cross-ring cleavage of the glycosyl moieties. In negative ion CID mass spectra aglycones of flavone di-*C*-glycosides were annotated based on the characteristic ion [aglycone + 2C_2_H_2_O-H]^−^ which was formed after ^0,2^X cross-ring cleavage of both glycosyl moieties. Aglycones of flavone/flavonol *O*-glycosides were identified in positive ion pseudo-MS^3^ experiments by fragmentation of protonated aglycone ions whose formation was induced by in-source fragmentation. The obtained spectra were referenced against CID mass spectra obtained from [M+H]^+^ ions of authentic flavone/flavonol standards.

#### Lignans

Like many other cereal grains (Smeds et al. 2007) wheat grains accumulate a structural diverse set of lignans of which 20 were annotated (see Supplemental Table 7e for analytical data). These comprise dibenzylbutyrolactone-type (L1–L4), furofurano-type (L5–L7) and furano-type lignans (L8) which partly occur as glycoconjugates, as well as sesqui- (L9–L12) and dilignans (L13). Due to their low polarity the annotated lignans eluted at high retention times (t_R_ 8.3–13.3 min). They were electrospray ionized in both ion modes predominantly forming [M+Na]^+^ and [M−H]^−^ ions, respectively. Under the applied analytical conditions ionization efficiency was found to be higher in the positive ion mode. However, for structure elucidation CID of [M−H]^−^ ions provided more informative mass spectra. The annotated dibenzylbutyrolactone-type lignans comprise a hydroxylated matairesinol derivative (L1), its hexose conjugate (L2) and corresponding methoxy homologs (L3, L4). Upon CID, [M−H]^−^ ions of L1 and L3 displayed a characteristic neutral loss of carbon dioxide due to cleavage of the lactone ring structure (Hanhineva et al. 2012). It was followed by elimination of substituted benzyl moieties, formaldehyde and methyl radicals. Syringaresinol (L5), a hydroxylated syringaresinol derivative (L6) and a syringaresinol hexose conjugate (L7) represent annotated furofurano-type lignans. Under CID conditions the [M−H]^−^ ion of L5 showed subsequent losses of two methyl radicals and formation of characteristic fragment ions at *m/z* 181.051 and *m/z* 166.027 which were formed by ^2,5^X cross-ring cleavage of a tetrahydrofuran ring and loss of a methyl radical (Morreel et al. 2010). Due to hydroxylation of the tetrahydrofuran ring system, the hydroxylated syringaresinol derivative L6 exhibited a somewhat different fragmentation behavior. Upon CID, the [M−H]^−^ ion of L6 showed subsequent neutral losses of a methyl radical and two molecules of formaldehyde. The fragment ion detected at *m/z* 166.027 represents the formal product of a ^2,5^X cross-ring cleavage starting from [M−H−CH_3_]^·−^. The furano-type lignan L8 was annotated as hexose conjugate of a dimethoxy homolog of lariciresinol. CID of the deprotonated molecular ion of L8 resulted in initial elimination of a hexose moiety. The resulting fragment ion [M−H−C_6_H_12_O_6_]^−^ showed subsequent losses of formaldehyde and two methyl radicals as well as loss of a dimethoxy quinone methide moiety (C_9_H_10_O_3_). Sesqui- and dilignans L9−L13 are composed of coniferyl (G) and sinapyl (S) alcohol monomers, which are connected via 8-*O*-4-(β-aryl ether type), 8-5-(phenylcoumaran type) and 8-8-(resinol type) linkage motifs. Molecular structures of L9−L13 can be elucidated by interpretation of CID mass spectra obtained from [M–H]^−^ ions using the published fragmentation pathways (Morreel et al. 2010). In case of sesquilignan L9, the sequence could not be unequivocally determined. Three isomers with similar CID mass spectrum were chromatographically separated for dilignan L13. For sesquilignans L9–L12, two isomers were resolved, which probably represent stereoisomers with different relative configuration (threo/erythro) of the 8-*O*-4-linkage.

#### Further phenolic metabolites

In addition to hydroquinones, hydroxycinnamic acid amides, flavonoids and lignans another 29 metabolites accumulating in wheat grains were annotated as phenolic metabolites (see Supplemental Table 7f for analytical data). These comprise hydroxybenzaldehydes (P1–P4), unconjugated hydroxybenzoic (P5–P9) and hydroxycinnamic acids (P10−P12) as well as coumarins (P21–P22). Hydroxybenzoic and hydroxycinnamic acids were nearly exclusively detected via their [M−H]^−^ ions in negative ion mode. In case of caffeic (P11) and ferulic acid (P12) geometrical isomers were chromatographically separable. Hydroxybenzaldehydes and coumarins formed quasimolecular ions upon electrospray ionization in both ion modes. Methoxy-subsititued derivatives such as vanillin (P3), syringaldehyde (P4) and scopoletin (22) were more efficiently ionized in positive ion mode. Due to their good commercial availability, aldehydes P1–P4, acids P5–P12 and coumarins P21–P22 were identified using authentic reference compounds. Beside these primitve phenolic metabolites, ferulic acid conjugates (P13–P17) and diferulic acids (P18–P20) were annotated. Under the applied analytical conditions ferulic acid conjugates P13–P17 were exclusively detected in positive ion mode forming [M+Na]^+^ ions and abundant in-source fragment ions at *m/z* 177.055. Formation of characteristic sodiated Y_1_ and B_2_ fragment ions following CID of [M+Na]^+^ ions suggested that P13 represents a conjugate of ferulic acid and a dipentoside. Conjugates P14−P17 could represent ferulic acid esters. Unfortunately, CID mass spectra obtained from [M+Na]^+^ ions of P14−P17 were less informative and only allowed determination of the elemental composition of the alcohol component. Elemental compositions and CID mass spectra of P18−P20 were consistent with didehydroferulic acid (P18) and diferulic acid (P19 and P20). Due to the large number of different linkage motifs described for didehydroferulic acids (Vismeh et al. 2013) and lack of comprehensive reference CID mass spectra linkage motifs in P18−P20 could not be determined.

#### Other metabolites

Besides secondary metabolites numerous polar and semi-polar primary metabolites were detected in hydroethanolic extracts of whole wheat grains and annotated (see Supplemental Tables 7g–j for analytical data). These comprise nucleobases and nucleosides (N1–N17), amino acids and derivatives such as amino acid betains, glycosylated amino acids, γ-glutamyl dipeptides (AA1–39), α-hydroxy acids, α,ω-dicarboxylic acids, di- to pentasaccharides, sugar acids (AS1−17), B vitamins and derivatives (V1−10). Most of the polar primary metabolites were poorly retained under the applied chromatographic conditions and eluted close to the void time without significant separation. Presence of hydrophobic moieties increased retention on a reversed phase and resulted in acceptable retention times for primary metabolites such as nucleosides, aliphatic and aromatic amino acids and their derivatives. Primary metabolites carrying easily protonable amino or quarternary ammonium groups were preferentially detected in positive ion mode and dominated the first quarter of the chromatogram. In contrast, α-hydroxy acids, α,ω-dicarboxylic acids, saccharides and sugar acids were more efficiently ionized in negative ion mode and became detectable as dominant peaks in the front part of the chromatogram in this ion mode.

### Method validation

#### Recovery rates and matrix effects

**Fig. 3:**
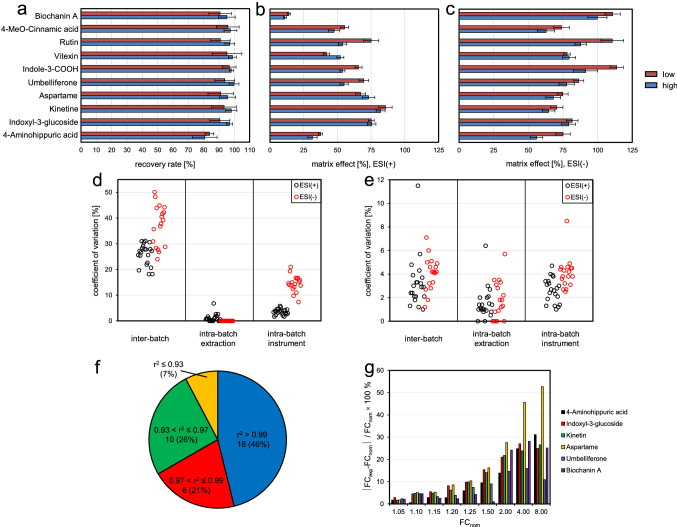
Method validation data. **a** Recovery rates of 10 model compounds spiked at two concentrations (low, high). Mean ± standard deviation (n = 4) is shown. **b** Effect of the sample matrix on the ionization efficiency of 10 model compounds spiked at two concentrations (low, high) in positive ion mode. Ion suppression: matrix effect < 100%; ion enhancement: matrix effect > 100%. Mean ± standard deviation (n = 4) is shown. **c** Effect of the sample matrix on the ionization efficiency of 10 model compounds spiked at two concentrations (low, high) in negative ion mode. Mean ± standard deviation (n = 4) is shown. **d** Repeatability data of the abundance of 39 metabolites without signal drift correction and normalization. **e** Repeatability data of the abundance of 39 metabolites with signal drift correction and normalization. **f** Linearity of the detector response upon extract dilution determined for 39 metabolites. **g** Accuracy of relative quantification of 6 model compounds spiked at different concentrations. FC_nom_, nominal fold-change; FC_exp_, experimentally determined fold-change

To validate the established metabolite profiling method recovery rates and matrix effects were determined for ten model compounds which were selected to be evenly distributed throughout the chromatogram and to represent major metabolite classes accumulating in wheat grains (Supplemental Table 2). Two individual spiking concentrations were used of which one was at the lower end of the model compound’s linear working range, the other one one order of magnitude higher. For all model compounds, good to excellent recovery rates were found without significant differences between the two spiking concentrations (Fig. 3a). Recovery rates of nine out of ten model compounds were found to be ≥ 90%. For the most polar one (4-amino hippuric acid) the recovery rate was 84% for the low and 81% for the high concentration. Matrix effects were determined in both ion modes (Fig. 3b, c). In positive ion mode the sample matrix reduced the electrospray ionization efficiency of all model compounds. At the high concentration weak ion suppression (< 25%) was detected for two, medium (> 25%, < 50%) for six and strong ion suppression (> 50%) for two test compounds, namely 4-amino hippuric acid and biochanin A. At the low concentration ion suppression effects were either equal or slightly different. 4-Amino hippuric acid (t_R_ 1.6 min) eluted in the front part of the chromatogram in which many abundant, readily ionizable metabolites such as amino acids and amino acid betains co-eluted and competed for charge during electrospray ionization. In case of biochanin A (t_R_ 15.6 min) strong ion suppression was induced by co-eluting proteins which started to elute in the rear part of the chromatogram (Supplemental Fig. 5f) and were partly detected as ion series with different charge in the higher *m/z* range (*m/z* 1000–1700). In the negative ion mode, ion suppression effects were less pronounced. At the high concentration weak ion suppression (< 25%) was now observed for six and medium ion suppression for the remaining four test compounds. Notably, the strong ion suppression effect found for biochanin A in positive ion mode was completely absent in negative ion mode. In contrast, ion suppression of 4-amino hippuric acid was only moderately decreased when changing the ion mode from positive to negative. This indicates, that in the front part of the chromatogram medium to strong ion suppression effects can also be expected to occur in negative ion mode.

#### Repeatability

To assess the repeatability of the whole analytical process a whole grain homogenate was repeatedly analyzed in five batches within a time period of two weeks. Abundances of 39 representative metabolites were evaluated (21 in positive and 18 in negative ion mode) without and with signal drift correction and normalization. Abundance patterns of two metabolites are exemplarily shown in Supplemental Fig. 7. According to the hierarchical design of the repeatability experiment the abundance of each metabolite was modelled by a nested linear random-effects model (Supplemental Table 4). Based on this model inter-batch and intra-batch technical variation were estimated for each metabolite (Fig. 3d, e; Supplemental Table 3).

Without signal drift correction and normalization the major fraction of variation observed within batches can be attributed to instrument variation (mean CV 8.6%) rather than to extraction variation (mean CV 0.6%). When looking at abundance patterns of individual metabolites a systematic decrease of the instrument’s sensitivity was observed within individual batches. This effect was more pronounced in negative than in positive ion mode (Fig. 3d) and can be probably attributed to a gradual contamination of the ion source and the front part of the ion optics during analysis by co-extracted proteins of which - despite a final protein precipitation step-residual amounts were present in the extracts. After prolonged analysis time (> 100 samples) contamination of the interior of the ion source became visible as thin white film, which can be easily wiped off during ion source maintenance. Inter-batch variation (mean CV 31.2%) exceeded intra-batch variation and was by far the largest source of variation without signal drift correction and normalization. The experimental design did not allow decomposition of inter-batch variation into instrument and extraction variation. However, relation of metabolite abundances in QC samples (which were prepared from samples of the first batch) to metabolite abundances in samples of batch two to five suggested that inter-batch variation was mainly caused by instrument variation. The high inter-batch instrument variation might be explained by changes of the sensitivity of the mass spectrometer during the two-week experiment which were partly due to full maintenance operations (ion source cleaning and instrument tuning) at the beginning of each batch.

To correct for instrument variation a QC sample-based signal drift correction and normalization approach was applied for each metabolite in each batch. Using this approach intra-batch instrument variation was significantly decreased (mean CV 3.3%, Fig. 3e) and only slightly higher than intra-batch extraction variation (mean CV 1.6%). Due to the normalization of metabolite abundances in samples to that of QC samples inter-batch variation was dramatically reduced (mean CV 3.6%) as well. Total technical variation after signal drift correction and normalization was in the range of 1.5–12.7% (mean CV 5.2%) which is well below the acceptance criteria of CV < 20% for LC/MS-based metabolite profiling studies (Dunn et al. 2012).

#### Linearity

Linearity was assessed for the same set of metabolites used in the repeatability experiment. Due to the very limited availability of reference compounds and analyte-free matrix or stable isotope labelled reference compounds serially diluted extracts were analyzed (Supplemental Table 5). A total of 21 out of 39 tested metabolites exhibited linear dilution curves within the whole concentration range tested. Due to detector saturation dilution curves of 4 metabolites were flattened in the higher concentration range. For the remaining 14 metabolites deviation from linearity could not be attributed to detector saturation. Here, other effects such as ion source saturation or ion suppression by charge competition between analyte and matrix might be reasons for the observed non-linear detector response. For 4 metabolites eluting in the front part of the chromatogram linearity of dilution curves was severely impaired reducing the linear working range to only one order of magnitude. This indicates, that relative quantification of metabolites eluting in this part of the chromatogram which is characterized by numerous incompletely separated highly abundant metabolites and subject of strong matrix effects has to be interpreted with caution. The coefficient of determination was calculated within the estimated linear working using a linear calibration model (Fig. 3f). Out of 39 tested metabolites, 3 showed poor (r^2^ ≤ 0.93), 10 moderate (0.93 < r^2^ ≤ 0.97), 8 good (0.97 < r^2^ ≤ 0.99) and 18 very good linearity (r^2^ > 0.99).

#### Accuracy of relative quantification

To evaluate the accuracy of relative quantification whole grain extracts were spiked with six test compounds at ten different concentrations and analyzed together with a pooled QC sample in three batches within a time period of one week. Peak areas of quantifier ions were determined and corrected batchwise for instrumental drift. In relation to the lowest concentration fold-changes were calculated from mean peak areas and tested for significance (Supplemental Table 6). At a 5% nominal concentration difference two-tailed Student’s *t*-test indicated a significant difference (df = 4, *P* ≤ 0.01) for mean peak areas of 4 out of 6 test compounds. At nominal concentration differences of 10% and 15% 5 out 6 test compounds differed significantly in abundance. At higher nominal concentration differences (≥ 20%) mean peak areas of all test compounds were found to be significantly different. This indicated, that a concentration difference of 15–20% can be robustly detected in multi-batch experiments after instrumental drift correction using a comparably low number of technical replicates (n = 3). However, prerequisite for this is that the compound to be relatively quantified is detected within the linear working range of the mass spectrometer. Relative errors of experimentally determined fold-changes were on average increasing with increasing nominal fold-change (Fig. 3g). For nominal concentration differences between 5 and 50% acceptable relative errors (average 5.7%, range 1–16%) were observed for experimentally determined fold-changes. At higher nominal concentration differences (200–800%) relative errors of fold-changes exceeded 20% in the majority of cases (average 26%, range 14–46%), although all concentrations of spiked test compounds were within the previously determined linear working range. One reason for the lower accuracy of high fold-changes might be caused by small systematic deviations in detector linearity leading to systematic errors in fold-change determination which significantly increase when analyzing samples with high concentration differences.

### Metabolite profiling

**Fig. 4:**
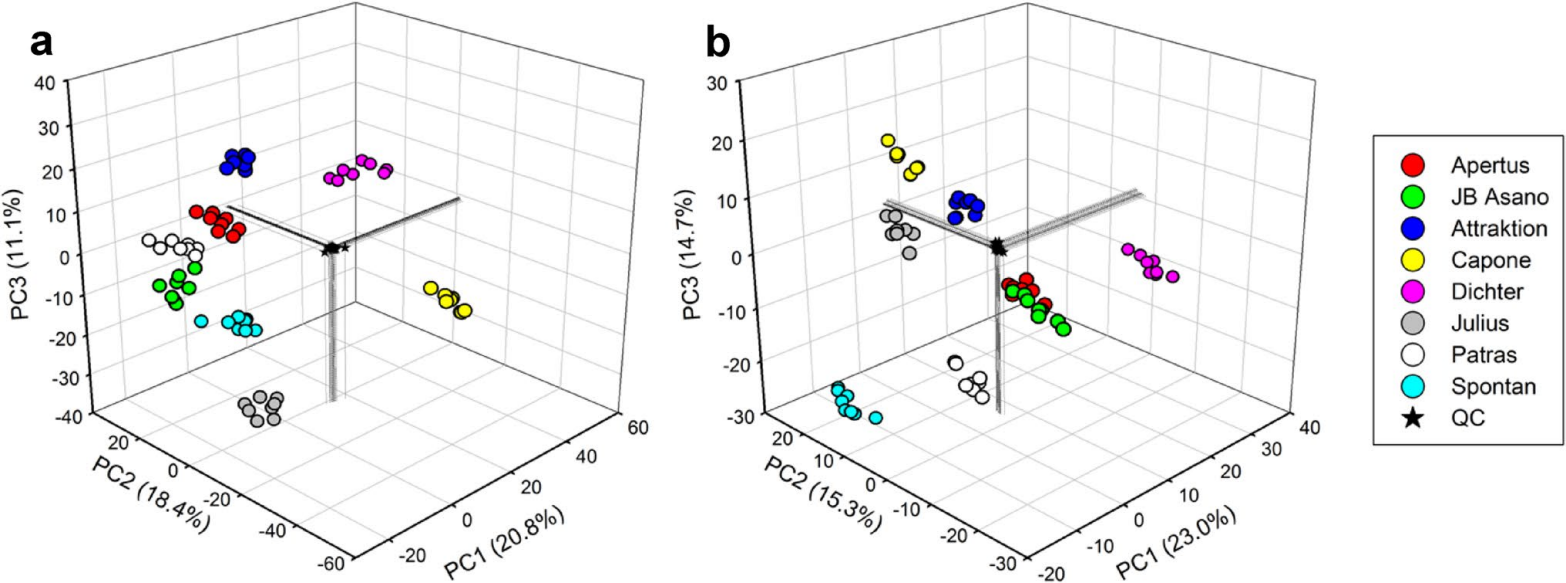
Non-targeted analysis of metabolite profiles. Metabolite profiles were obtained from 64 hydroethanolic whole wheat grain extracts (8 cultivars × 4 agronomic replicates × 2 technical replicates) using UHPLC/ESI-QTOFMS in both ion modes. A quality control (QC) sample was prepared by pooling aliquots of all extracts and repeatedly injected 19 times within the batch. **a** Principal component analysis based on drift-corrected and normalized abundances of 4155 molecular features detected in positive ion mode. The score plot of the first three principal components explaining 50.3% of the total variance is shown. **b** Principal component analysis based on drift-corrected and normalized abundances of 1886 molecular features detected in negative ion mode. The score plot of the first three principal components explaining 53.0% of the total variance is shown

To demonstrate the applicability of the analytical approach for metabolite profiling whole grain samples of eight soft winter wheat cultivars (‘Apertus’, ‘Attraktion’, ‘Capone’, ‘Dichter’, ‘JB Asano’, ‘Julius’, ‘Patras’, ‘Spontan’) obtained from a one-year field trial with four agronomic replicates were extracted in duplicate and analyzed together with a pooled QC sample by UHPLC/ESI-QTOFMS in positive and negative ion mode. For non-targeted data analysis, molecular features were extracted from raw data and aligned using the XCMS algorithm. After elimination of molecular features with low repeatability in pooled QC samples two data sets comprising 4155 and 1886 molecular features were obtained from raw data acquired in positive and negative ion mode, respectively. Feature intensities were normalized by sample weight, corrected for instrumental drift and normalized using the pooled QC sample, log_2_-transformed and subjected to principal component analysis. The score plots of the first three principle components (PCs, Fig. 4) explaining more than 50% of the total variance of both data sets show a highly cultivar-specific discrimination of biological samples while pooled QC samples were tightly clustered in the center. In both data sets PC1 strongly discriminated samples of ‘Dichter’ from samples of the other cultivars. In contrast, discrimination of cultivars along PC2 and PC3 was different in both data sets. The cultivar-specific clustering observed in both score plots was also reflected by a high number of molecular features with cultivar-dependent differences in intensity. As revealed by *one-way* ANOVA, intensities of 78% (85%) of the molecular features detected in positive (negative) ion were significantly affected by cultivar (*FDR* ≤ 0.01).

**Fig. 5:**
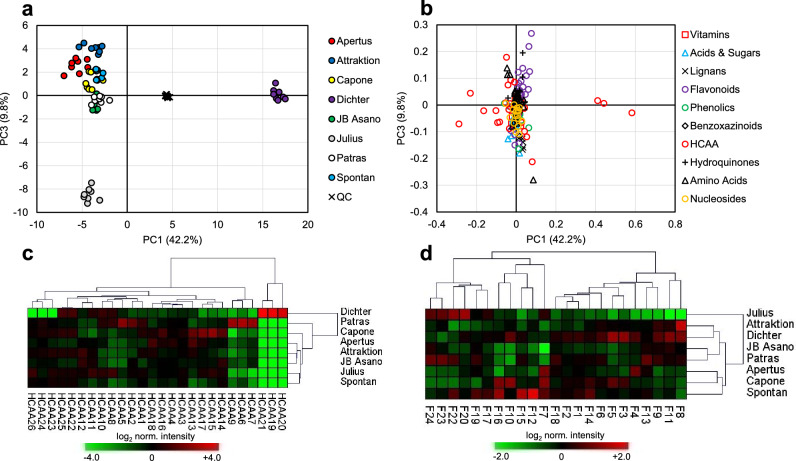
Targeted analysis of metabolite profiles. Metabolite profiles were obtained from 64 hydroethanolic whole wheat grain extracts (8 cultivars × 4 agronomic replicates × 2 technical replicates) using UHPLC/ESI-QTOFMS in both ion modes. A quality control (QC) sample was prepared by pooling aliquots of all extracts and repeatedly injected 19 times within the batch. **a** Principal component analysis based on drift-corrected and normalized abundances of 167 metabolites (isomers combined). The score plot of the first and the third principal component explaining 52.0% of the total variance is shown. **b** Corresponding loading plot. **c** Heatmap representation of normalized abundances of hydroxycinnamic acid amides and hierarchical cluster analysis (Euclidean distance, average linkage method). Agronomic and technical replicates were averaged. **d** Heatmap representation of normalized abundances of flavonoids and hierarchical cluster analysis (Euclidean distance, average linkage method). Agronomic and technical replicates were averaged. For compound labelling see Supplemental Table 7b and d

To gain a deeper insight into metabolic differences between cultivars a targeted analysis of the annotated metabolites was performed. Metabolites detected with low intensity and low repeatability in pooled QC samples were excluded from further analysis and isomeric metabolites combined. Intensities of the resulting 167 metabolites were normalized by sample weight, corrected for instrumental drift and normalized using the pooled QC samples, log_2_-transformed and subjected to principal component analysis. Pairwise score and loading plots of the first three PCs explaining 67% of the total variance are given in Fig. 5 and Supplemental Fig. 8. As observed in non-targeted data analyses, PC1 strongly discriminated samples of ‘Dichter’. As indicated in the loading plots, HCAAs were essentially responsible for this discrimination along PC1 but also provided significant contributions to PC2 and PC3. Hierarchical cluster analysis and heatmap visualization of HCAA abundance profiles (Fig. 5c) revealed that in comparison to other cultivars grains of ‘Dichter’ accumulated high levels of diacylated spermine conjugates containing a feruloyl moiety (HCAA19, 20, 21). In contrast, levels of homologous conjugates containing a 3,4-dimethoxycinnamoyl moiety instead of a feruloyl moiety (HCAA23, 24, 26) were significantly lower in grains of ‘Dichter’ compared to the other cultivars. Similarily, oxygenated agmatine conjugates (HCAA6, 7, 9) accumulated in comparable high levels in grains of ‘Patras’, whereas diacylated spermine conjugates (HCAA14–17) displayed highest levels in grains of ‘Capone’. Besides HCAAs, also flavonoids significantly contribute to PC2 and in particular to PC3 which strongly discriminated samples of ‘Julius’ (Fig. 5a, b). Hierarchical cluster analysis and heat map visualization of flavonoid abundance profiles (Fig. 5d) revealed a distinct chemotype of ‘Julius’ which is characterized by medium to low levels of flavone glycosides (F1–19) and high levels of flavonol glycosides (F20–24). Hierarchical cluster analyses and heatmap visualization of the abundance profiles of other metabolite classes are given in Supplemental Fig. 9.

## Concluding remarks

Based on reversed-phase UHPLC/ESI-QTOFMS an analytical workflow for comprehensive profiling of semi-polar phytochemicals from whole wheat grains was developed and validated in this study. The workflow primarily targets semi-polar secondary metabolites such as hydroquinones, hydroxycinnamic acid amides, flavonoids, benzoxazinoids, lignans and other phenolics. In addition, numerous primary metabolites can be detected including nucleosides, amino acids, amino acid betains, organic acids, saccharides and B vitamin derivatives. Most of them elute in the front part of the chromatogram without sufficient chromatographic separation which complicates their relative quantification due to strong matrix effects. To separate and to reliably quantify these polar metabolites orthogonal chromatographic approaches have to be applied.

Based on literature searches, database queries, analyses of reference compounds and *de novo* interpretation of accurate mass tandem mass spectra a total of 248 metabolites were annotated in this study. The analytical data compiled represent a useful resource for targeted metabolite profiling of whole wheat grains. It remains to be tested whether such a targeted data analysis approach can be used for class-specific quantification of metabolites. In addition, the collected CID mass spectra should facilitate annotation of further representatives of the above mentioned metabolite classes when analyzing cultivars other than those used in this study.

Due to the presence of protein fractions soluble in aqueous organic solvents particular attention has to be paid to the choice of the extraction solvent. Use of ethanol-water, 4/1 (v/v) resulted in good to excellent recovery rates for acidic, neutral and basic semi-polar metabolites and suppressed enzymatic deglycosylation during extraction. Among nine solvent mixtures tested, ethanol-water, 4/1 (v/v) displayed at the same time an intermediate tendency for co-extraction of matrix components.

Despite an additional protein precipitation step, residual amounts of protein and other matrix components are present in the final extract, which contaminate the ESI interface during analysis and induce a significant decrease in the sensitivity of the mass spectrometer within analytical batches. To correct for this effect in an analyte-specific manner a pooled QC sample-based signal drift correction and normalization approach was applied. This approach significantly reduced intra- and inter-batch instrument variability and resulted in good repeatabilities (mean CV 5.2%) of the whole analytical workflow.

Strong ion suppression effects were detected in both ion modes in the front part of the chromatogram in which many poorly separated abundant primary metabolites elute. In positive ion mode, matrix effects were additionally observed in the rear part of the chromatogram due to co-eluting gliadin proteins. For metabolites eluting in both chromatographic regions relative quantification should be handled with caution and validated by other analytical methods when possible.

Using the QC sample-based signal drift correction and normalization approach concentration differences of 15–20% can be robustly detected between two samples in multi-batch experiments with three technical replicates. For smaller concentration differences accuracy of relative quantification was good (mean relative error 5.7%), whereas larger relative errors > 20% must be expected for higher concentration differences.

In conjunction with non-targeted and targeted data analysis strategies the developed analytical workflow was successfully applied to discern differences in the profiles of semi-polar secondary metabolites in whole grain samples of eight winter wheat cultivars. Compared to analytical methods targeting only single compound classes, this analytical approach provides a more comprehensive view on the complex metabolic profile of wheat grains and represents therefore a powerful tool to catalogue their metabolic diversity dependent on genetic and environmental factors.

## Supplementary Information

Below is the link to the electronic supplementary material.

Electronic supplementary material 1 (PPTX 2600 kb)

Electronic supplementary material 2 (DOCX 50 kb)

Electronic supplementary material 3 (XLSX 82 kb)

## Data Availability

Raw metabolite profiles and corresponding metadata are available from the MetaboLights repository under the accession number MTBLS 2276.
