# Supplementary material for: Comprehensive profiling of semi‐polar phytochemicals in whole wheat grains (Triticum aestivum) using liquid chromatography coupled with electrospray ionization quadrupole time‐of‐flight mass spectrometry
Source: Metabolomics. 2021 Jan 27;17(2):18. doi: 10.1007/s11306-020-01761-4 (PMC7840630; doi:10.1007/s11306-020-01761-4)
Supplement: Supplementary file 1 — Electronic supplementary material 1 (PPTX 2600 kb) [file 11306_2020_1761_MOESM1_ESM.pptx]

## Slide 1
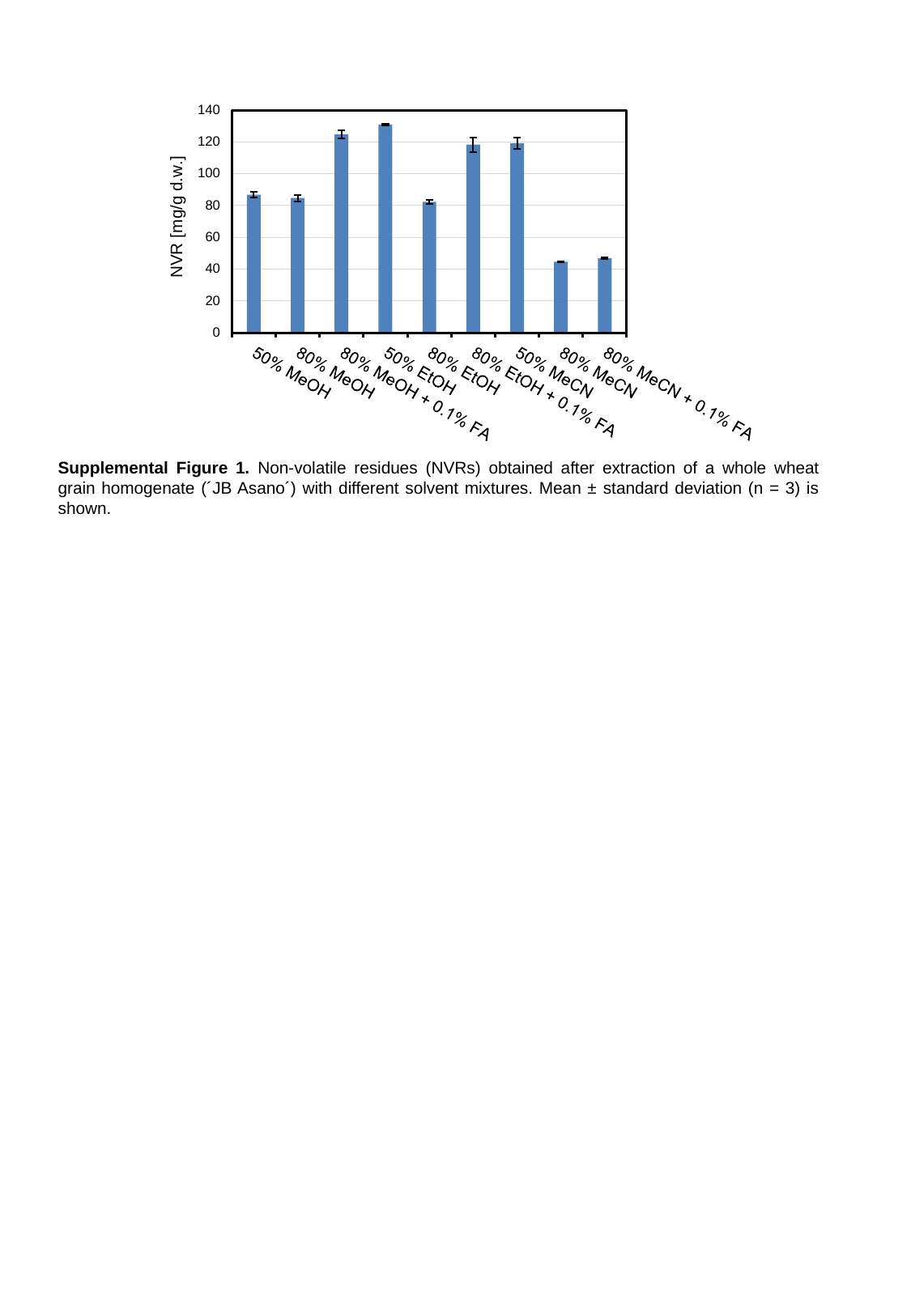

Supplemental Figure 1. Non-volatile residues (NVRs) obtained after extraction of a whole wheat grain homogenate (´JB Asano´) with different solvent mixtures. Mean ± standard deviation (n = 3) is shown.

## Slide 2
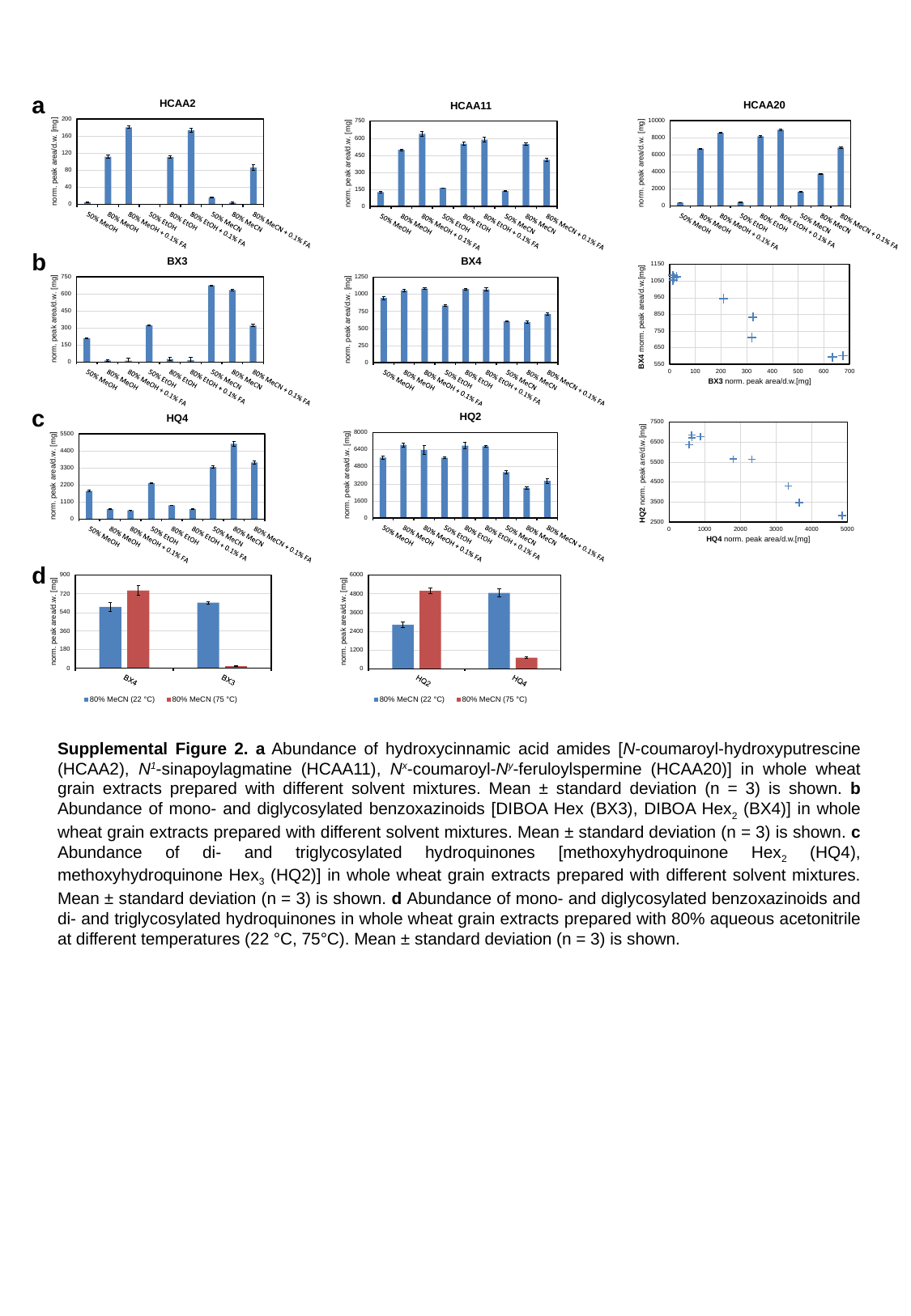

a
b
c
d
Supplemental Figure 2. a Abundance of hydroxycinnamic acid amides [N-coumaroyl-hydroxyputrescine (HCAA2), N1-sinapoylagmatine (HCAA11), Nx-coumaroyl-Ny-feruloylspermine (HCAA20)] in whole wheat grain extracts prepared with different solvent mixtures. Mean ± standard deviation (n = 3) is shown. b Abundance of mono- and diglycosylated benzoxazinoids [DIBOA Hex (BX3), DIBOA Hex2 (BX4)] in whole wheat grain extracts prepared with different solvent mixtures. Mean ± standard deviation (n = 3) is shown. c Abundance of di- and triglycosylated hydroquinones [methoxyhydroquinone Hex2 (HQ4), methoxyhydroquinone Hex3 (HQ2)] in whole wheat grain extracts prepared with different solvent mixtures. Mean ± standard deviation (n = 3) is shown. d Abundance of mono- and diglycosylated benzoxazinoids and di- and triglycosylated hydroquinones in whole wheat grain extracts prepared with 80% aqueous acetonitrile at different temperatures (22 °C, 75°C). Mean ± standard deviation (n = 3) is shown.

## Slide 3
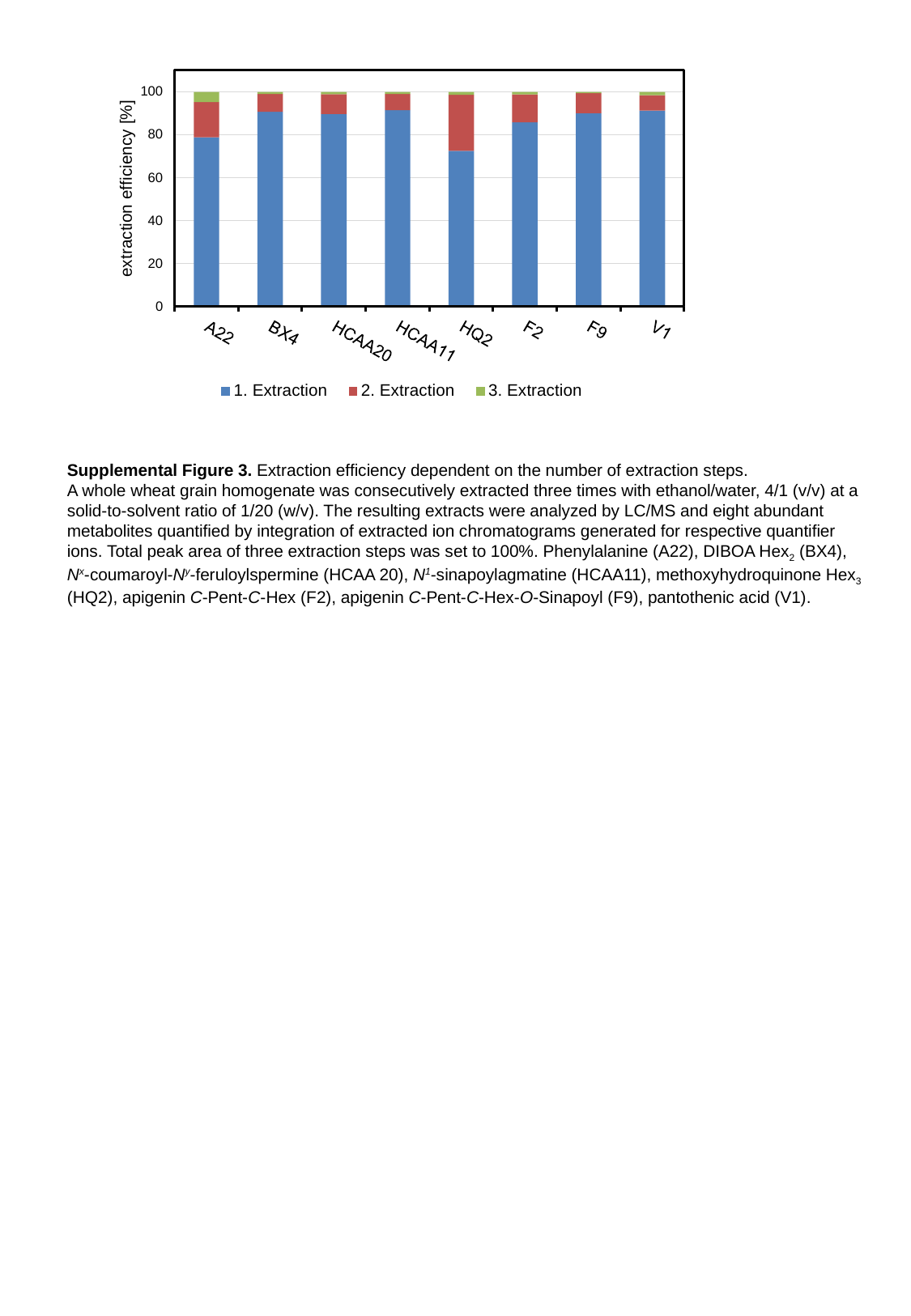

Supplemental Figure 3. Extraction efficiency dependent on the number of extraction steps.
A whole wheat grain homogenate was consecutively extracted three times with ethanol/water, 4/1 (v/v) at a solid-to-solvent ratio of 1/20 (w/v). The resulting extracts were analyzed by LC/MS and eight abundant metabolites quantified by integration of extracted ion chromatograms generated for respective quantifier ions. Total peak area of three extraction steps was set to 100%. Phenylalanine (A22), DIBOA Hex2 (BX4), Nx-coumaroyl-Ny-feruloylspermine (HCAA 20), N1-sinapoylagmatine (HCAA11), methoxyhydroquinone Hex3 (HQ2), apigenin C-Pent-C-Hex (F2), apigenin C-Pent-C-Hex-O-Sinapoyl (F9), pantothenic acid (V1).

## Slide 4
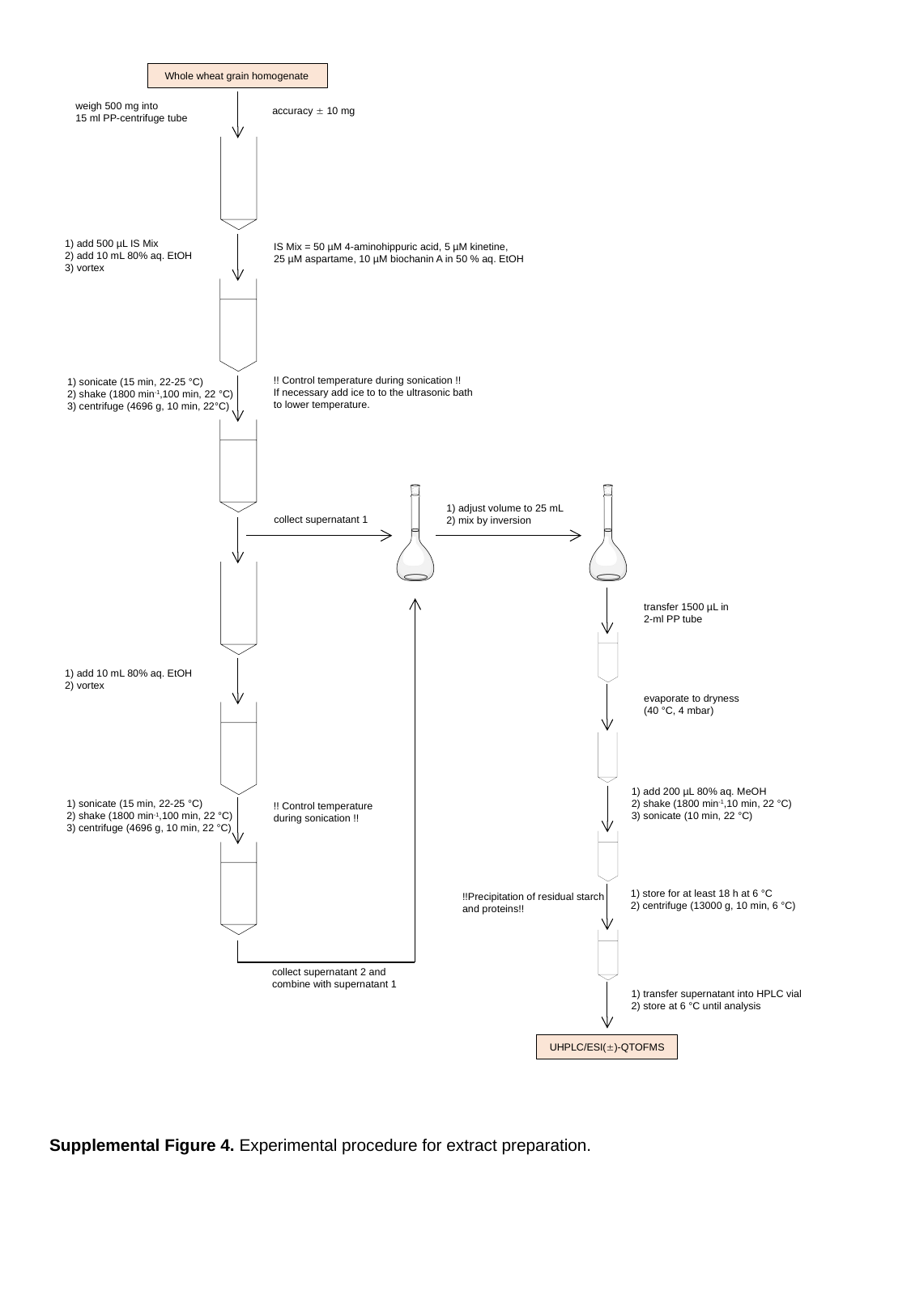

Whole wheat grain homogenate
weigh 500 mg into
15 ml PP-centrifuge tube
accuracy ± 10 mg
1) add 500 µL IS Mix
2) add 10 mL 80% aq. EtOH
3) vortex
IS Mix = 50 µM 4-aminohippuric acid, 5 µM kinetine,
25 µM aspartame, 10 µM biochanin A in 50 % aq. EtOH
!! Control temperature during sonication !!
If necessary add ice to to the ultrasonic bath
to lower temperature.
1) sonicate (15 min, 22-25 °C)
2) shake (1800 min-1,100 min, 22 °C)
3) centrifuge (4696 g, 10 min, 22°C)
1) adjust volume to 25 mL
2) mix by inversion
collect supernatant 1
transfer 1500 µL in
2-ml PP tube
1) add 10 mL 80% aq. EtOH
2) vortex
evaporate to dryness
(40 °C, 4 mbar)
1) add 200 µL 80% aq. MeOH
2) shake (1800 min-1,10 min, 22 °C)
3) sonicate (10 min, 22 °C)
1) sonicate (15 min, 22-25 °C)
2) shake (1800 min-1,100 min, 22 °C)
3) centrifuge (4696 g, 10 min, 22 °C)
!! Control temperature
during sonication !!
1) store for at least 18 h at 6 °C
2) centrifuge (13000 g, 10 min, 6 °C)
!!Precipitation of residual starch
and proteins!!
collect supernatant 2 and
combine with supernatant 1
1) transfer supernatant into HPLC vial
2) store at 6 °C until analysis
UHPLC/ESI(±)-QTOFMS
Supplemental Figure 4. Experimental procedure for extract preparation.

## Slide 5
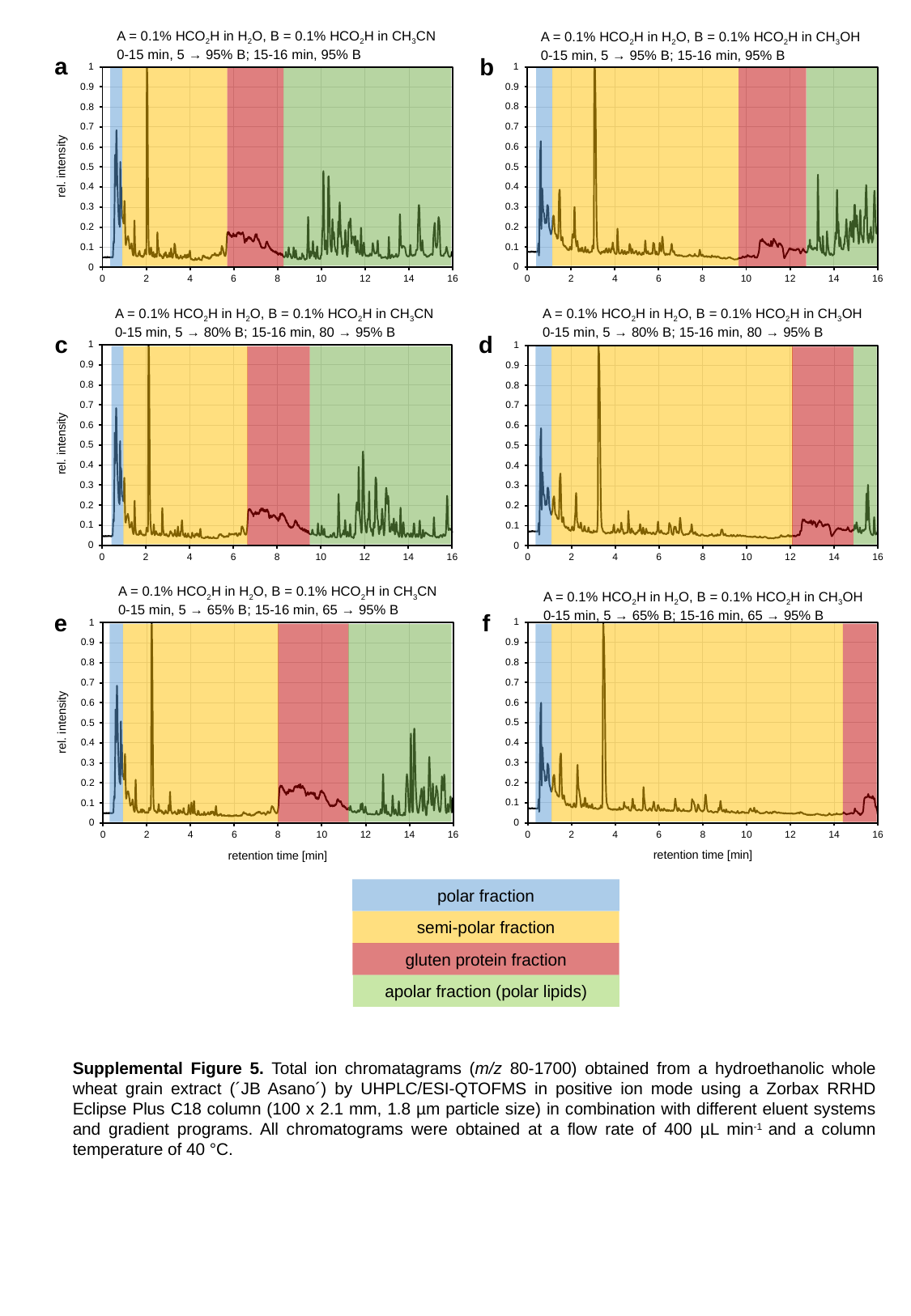

A = 0.1% HCO2H in H2O, B = 0.1% HCO2H in CH3CN
0-15 min, 5 → 95% B; 15-16 min, 95% B
A = 0.1% HCO2H in H2O, B = 0.1% HCO2H in CH3OH
0-15 min, 5 → 95% B; 15-16 min, 95% B
a
b
rel. intensity
A = 0.1% HCO2H in H2O, B = 0.1% HCO2H in CH3OH
0-15 min, 5 → 80% B; 15-16 min, 80 → 95% B
A = 0.1% HCO2H in H2O, B = 0.1% HCO2H in CH3CN
0-15 min, 5 → 80% B; 15-16 min, 80 → 95% B
d
c
rel. intensity
A = 0.1% HCO2H in H2O, B = 0.1% HCO2H in CH3CN
0-15 min, 5 → 65% B; 15-16 min, 65 → 95% B
A = 0.1% HCO2H in H2O, B = 0.1% HCO2H in CH3OH
0-15 min, 5 → 65% B; 15-16 min, 65 → 95% B
f
e
rel. intensity
retention time [min]
retention time [min]
polar fraction
semi-polar fraction
gluten protein fraction
apolar fraction (polar lipids)
Supplemental Figure 5. Total ion chromatagrams (m/z 80-1700) obtained from a hydroethanolic whole wheat grain extract (´JB Asano´) by UHPLC/ESI-QTOFMS in positive ion mode using a Zorbax RRHD Eclipse Plus C18 column (100 x 2.1 mm, 1.8 µm particle size) in combination with different eluent systems and gradient programs. All chromatograms were obtained at a flow rate of 400 µL min-1 and a column temperature of 40 °C.

## Slide 6
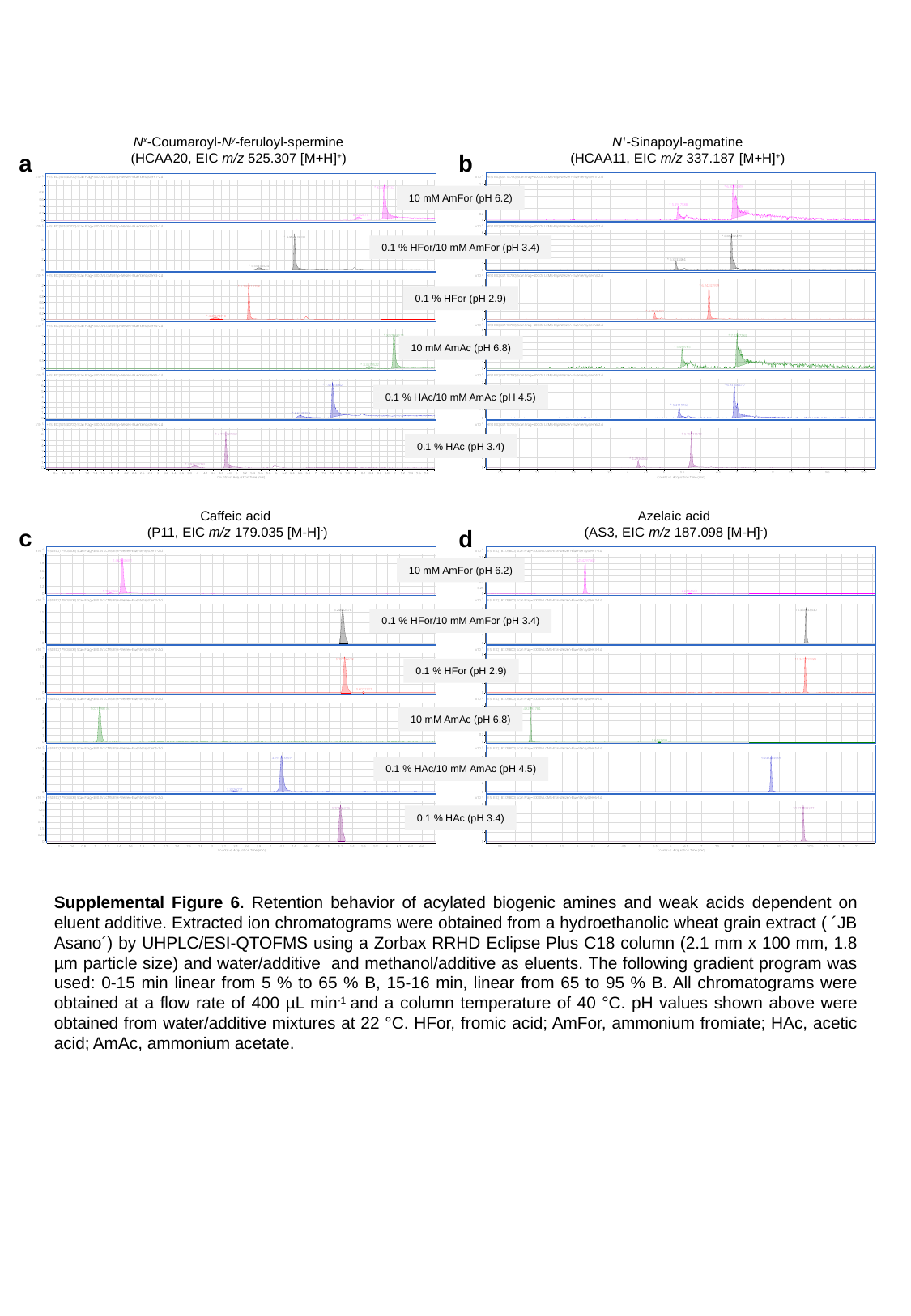

Nx-Coumaroyl-Ny-feruloyl-spermine
(HCAA20, EIC m/z 525.307 [M+H]+)
N1-Sinapoyl-agmatine
(HCAA11, EIC m/z 337.187 [M+H]+)
b
a
10 mM AmFor (pH 6.2)
0.1 % HFor/10 mM AmFor (pH 3.4)
0.1 % HFor (pH 2.9)
10 mM AmAc (pH 6.8)
0.1 % HAc/10 mM AmAc (pH 4.5)
0.1 % HAc (pH 3.4)
Caffeic acid
(P11, EIC m/z 179.035 [M-H]-)
Azelaic acid
(AS3, EIC m/z 187.098 [M-H]-)
c
d
10 mM AmFor (pH 6.2)
0.1 % HFor/10 mM AmFor (pH 3.4)
0.1 % HFor (pH 2.9)
10 mM AmAc (pH 6.8)
0.1 % HAc/10 mM AmAc (pH 4.5)
0.1 % HAc (pH 3.4)
Supplemental Figure 6. Retention behavior of acylated biogenic amines and weak acids dependent on eluent additive. Extracted ion chromatograms were obtained from a hydroethanolic wheat grain extract ( ´JB Asano´) by UHPLC/ESI-QTOFMS using a Zorbax RRHD Eclipse Plus C18 column (2.1 mm x 100 mm, 1.8 µm particle size) and water/additive and methanol/additive as eluents. The following gradient program was used: 0-15 min linear from 5 % to 65 % B, 15-16 min, linear from 65 to 95 % B. All chromatograms were obtained at a flow rate of 400 µL min-1 and a column temperature of 40 °C. pH values shown above were obtained from water/additive mixtures at 22 °C. HFor, fromic acid; AmFor, ammonium fromiate; HAc, acetic acid; AmAc, ammonium acetate.

## Slide 7
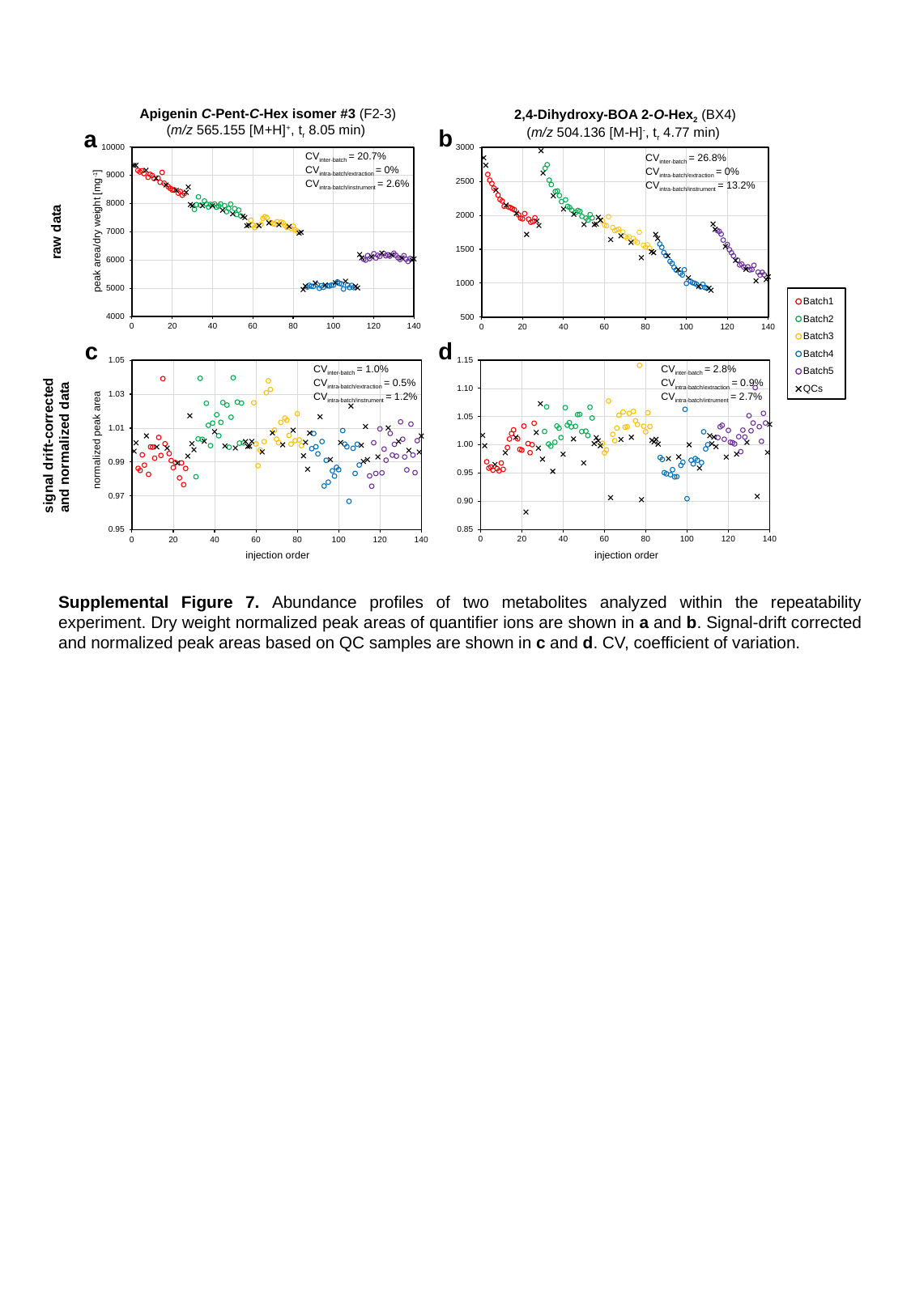

Apigenin C-Pent-C-Hex isomer #3 (F2-3)
(m/z 565.155 [M+H]+, tr 8.05 min)
2,4-Dihydroxy-BOA 2-O-Hex2 (BX4)
(m/z 504.136 [M-H]-, tr 4.77 min)
b
a
CVinter-batch = 20.7%
CVintra-batch/extraction = 0%
CVintra-batch/instrument = 2.6%
CVinter-batch = 26.8%
CVintra-batch/extraction = 0%
CVintra-batch/instrument = 13.2%
raw data
peak area/dry weight [mg-1]
c
d
CVinter-batch = 1.0%
CVintra-batch/extraction = 0.5%
CVintra-batch/instrument = 1.2%
CVinter-batch = 2.8%
CVintra-batch/extraction = 0.9%
CVintra-batch/intrument = 2.7%
signal drift-corrected
and normalized data
normalized peak area
injection order
injection order
Supplemental Figure 7. Abundance profiles of two metabolites analyzed within the repeatability experiment. Dry weight normalized peak areas of quantifier ions are shown in a and b. Signal-drift corrected and normalized peak areas based on QC samples are shown in c and d. CV, coefficient of variation.

## Slide 8
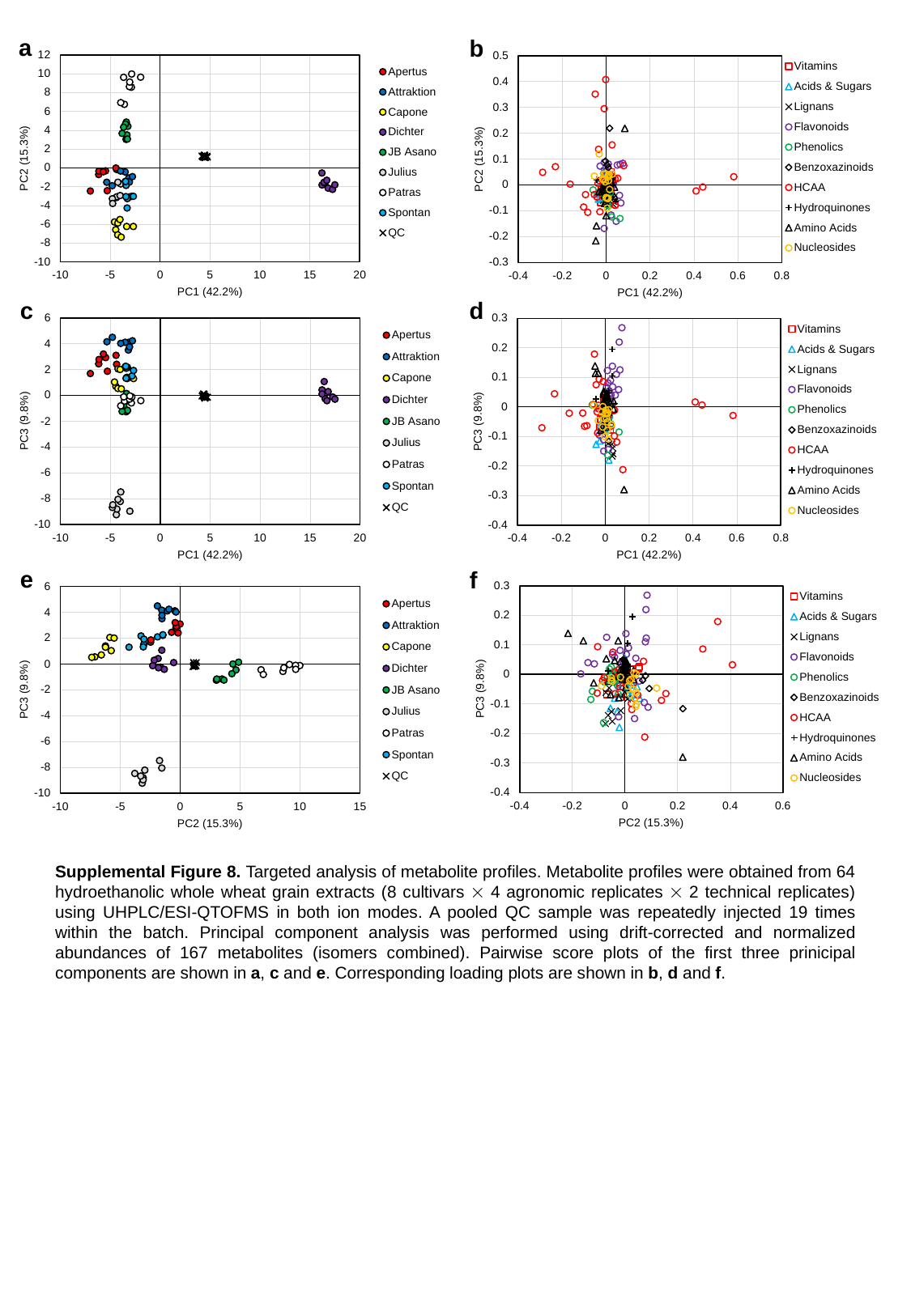

a
b
c
d
e
f
Supplemental Figure 8. Targeted analysis of metabolite profiles. Metabolite profiles were obtained from 64 hydroethanolic whole wheat grain extracts (8 cultivars × 4 agronomic replicates × 2 technical replicates) using UHPLC/ESI-QTOFMS in both ion modes. A pooled QC sample was repeatedly injected 19 times within the batch. Principal component analysis was performed using drift-corrected and normalized abundances of 167 metabolites (isomers combined). Pairwise score plots of the first three prinicipal components are shown in a, c and e. Corresponding loading plots are shown in b, d and f.

## Slide 9
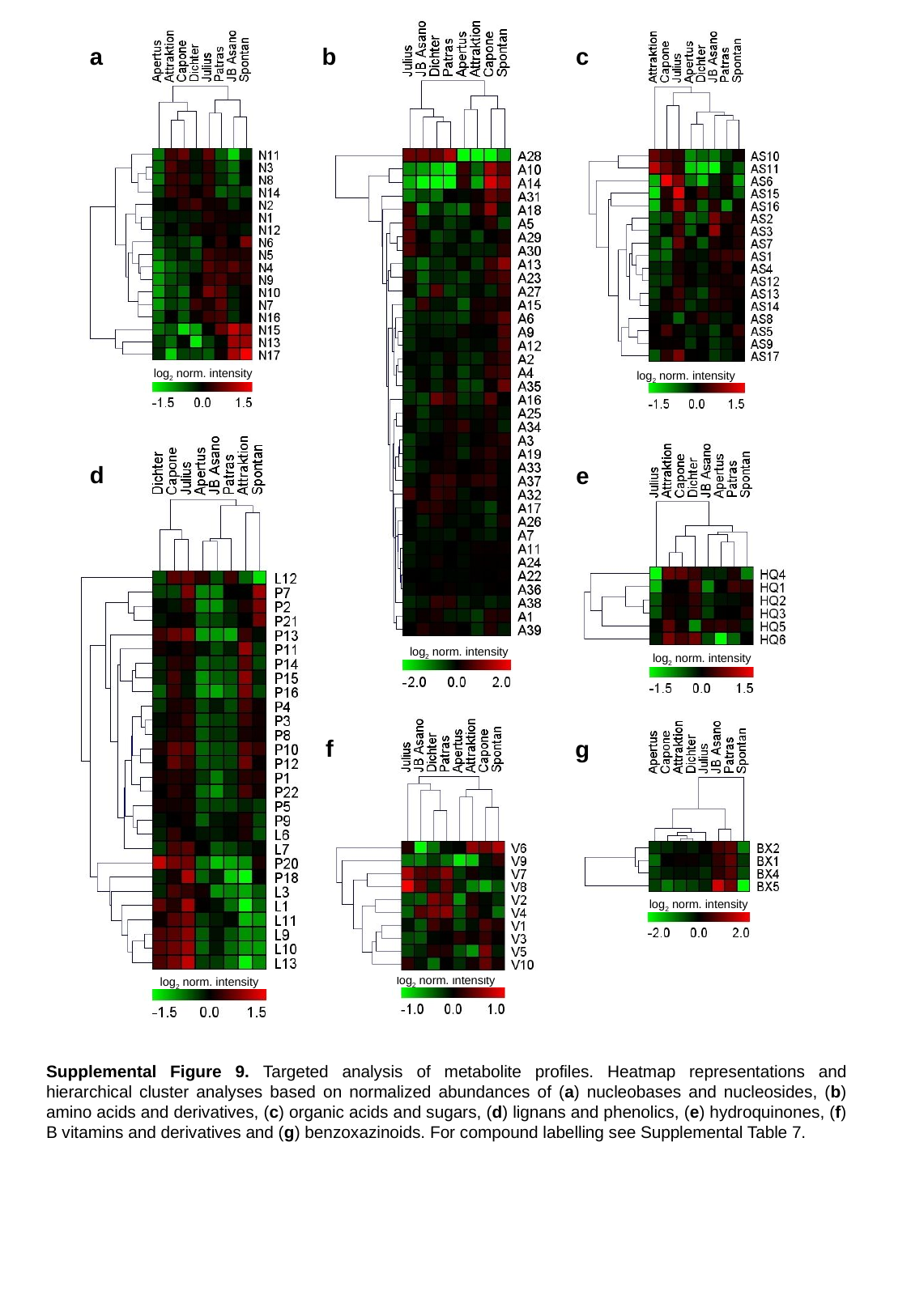

log2 norm. intensity
log2 norm. intensity
log2 norm. intensity
a
c
b
log2 norm. intensity
log2 norm. intensity
d
e
log2 norm. intensity
log2 norm. intensity
f
g
Supplemental Figure 9. Targeted analysis of metabolite profiles. Heatmap representations and hierarchical cluster analyses based on normalized abundances of (a) nucleobases and nucleosides, (b) amino acids and derivatives, (c) organic acids and sugars, (d) lignans and phenolics, (e) hydroquinones, (f) B vitamins and derivatives and (g) benzoxazinoids. For compound labelling see Supplemental Table 7.
