# Supplementary material for: Comprehensive profiling of semi‐polar phytochemicals in whole wheat grains (Triticum aestivum) using liquid chromatography coupled with electrospray ionization quadrupole time‐of‐flight mass spectrometry
Source: Metabolomics. 2021 Jan 27;17(2):18. doi: 10.1007/s11306-020-01761-4 (PMC7840630; doi:10.1007/s11306-020-01761-4)
Supplement: Supplementary file 2 — Electronic supplementary material 2 (DOCX 50 kb) [file 11306_2020_1761_MOESM2_ESM.docx]

**Supplemental Table 1.** Reference compounds used for authentication of metabolites, as model compounds for method validation or internal standards.

| **No.** | **Name** | **PubChem CID** | **Supplier** | **No.** | **Name** | **PubChem CID** | **Supplier** |
| --- | --- | --- | --- | --- | --- | --- | --- |
| **N1** | Adenosine | 60961 | Sigma-Aldrich | **P1** | 4-Hydroxybenzaldehyde | 126 | Sigma-Aldrich |
| **N7** | Guanosine | 6802 | Sigma-Aldrich | **P2** | Protocatechualdehyde | 8768 | Sigma-Aldrich |
| **N11** | Thymidine | 5789 | Sigma-Aldrich | **P3** | Vanillin | 1183 | Sigma-Aldrich |
| **N12** | Uridine | 6029 | TCI | **P4** | Syringaldehyde | 141513 | Fluka |
| **N13** | Xanthine | 1188 | TCI | **P5** | 4-Hydroxybenzoic acid | 135 | Fluka |
| **N14** | Xanthosine dihydrate | 64959 | TCI | **P6** | Salicylic acid | 338 | Sigma-Aldrich |
| **N15** | Hypoxanthine | 790 | Sigma-Aldrich | **P7** | Protocatechuic acid | 72 | Sigma-Aldrich |
| **N16** | Inosine | 6021 | Sigma-Aldrich | **P8** | Vanillic acid | 8468 | Fluka |
| **N17** | Uric acid | 1175 | TCI | **P9** | Syringic acid | 10742 | Fluka |
| **A1** | L-Aspartic acid | 5960 | Sigma-Aldrich | **P10** | p-Coumaric acid | 637542 | Fluka |
| **A2** | L-Asparagine | 6267 | Sigma-Aldrich | **P11** | Caffeic acid | 689043 | Fluka |
| **A3** | L-Glutamic acid | 33032 | TCI | **P12** | Ferulic acid | 445858 | Carl Roth GmbH |
| **A4** | L-Pyroglutamic acid | 499 | TCI | **P21** | Esculetin | 5281416 | Sigma-Aldrich |
| **A6** | L-Glutamine | 5961 | TCI | **P22** | Scopoletin | 5280460 | Sigma-Aldrich |
| **A7** | Glycine betaine | 247 | Sigma-Aldrich | **F21** | Rutin Trihydrate | 16218542 | Merck |
| **A8** | L-Valine | 6287 | Sigma-Aldrich | **F22** | Isorhamnetin-3-*O*-Rutinoside | 45933931 | Carl Roth GmbH |
| **A11** | L-Leucine | 6106 | TCI | **-** | Apigenin | 5280443 | Carl Roth GmbH |
| **A12** | L-Isoleucine | 6306 | TCI | **-** | Luteolin | 5280445 | Extrasynthese |
| **A15** | L-Proline | 145742 | TCI | **-** | Chrysoeriol | 5280666 | Extrasynthese |
| **A19** | L-Methionine | 6137 | Sigma-Aldrich | **-** | Kaempferol | 5280863 | Indofine |
| **A20** | Glutathione | 124886 | Sigma-Aldrich | **-** | Quercetin Dihydrate | 5284452 | Sigma |
| **A22** | L-Phenylalanine | 6140 | Sigma-Aldrich | **-** | Isorhamnetin | 5281654 | Carl Roth GmbH |
| **A24** | L-Tyrosine | 6057 | Fluka | **-** | Syringetin | 5281953 | Extrasynthese |
| **A26** | L-Tryptophan | 6305 | Sigma-Aldrich | **-** | 2-Methoxyhydroquinone | 69988 | Sigma-Aldrich |
| **A30** | Kynurenic acid | 3845 | Sigma-Aldrich | **-** | 2,6-Dimethoxyhydroquinone | 96038 | Sigma-Aldrich |
| **A31** | L-Arginine | 6322 | Sigma-Aldrich | **-** | 4-Aminohippuric acid | 2148 | Merck |
| **A33** | L-Lysine | 5962 | Sigma-Aldrich | **-** | 3-Indoxyl β-D-glucoside | 441564 | Sigma-Aldrich |
| **A36** | Choline chloride | 305 | TCI | **-** | Kinetin | 3830 | Sigma-Aldrich |
| **AS1** | Succinic acid | 1110 | Carl Roth GmbH | **-** | Aspartame | 134601 | Sigma-Aldrich |
| **AS2** | Suberic acid | 10457 | Sigma-Aldrich | **-** | Umbelliferone | 5281426 | Sigma-Aldrich |
| **AS3** | Azelaic acid | 2266 | Sigma-Aldrich | **-** | Indole-3-carboxylic acid | 69867 | Sigma-Aldrich |
| **AS4** | L-(-)-Malic acid | 525 | Acros | **-** | Vitexin | 5280441 | Sigma-Aldrich |
| **AS5** | Citric acid monohydrate | 22230 | AppliChem | **-** | 4-Methoxycinnamic acid | 699414 | Sigma-Aldrich |
| **AS7** | Quinic acid | 6508 | Sigma-Aldrich | **-** | Biochanin A | 5280373 | Sigma-Aldrich |
| **V1** | Sodium D-pantothenate | 23679004 | TCI |  |  |  |  |
| **V3** | Thiamine hydrochloride | 6202 | Sigma-Aldrich |  |  |  |  |
| **V5** | Trigonelline hydrochloride | 134606 | Sigma-Aldrich |  |  |  |  |
| **V6** | Methylnicotinate | 7151 | Sigma-Aldrich |  |  |  |  |
| **V8** | Nicotinic acid | 938 | Sigma-Aldrich |  |  |  |  |

**Supplemental Table 2.** Linear range, recovery rates and matrix effects determined for ten model compounds.

| **test**  **compound** | **retention**  **time**  *[min]* | **quantifier Ion** | | **linear range**  *[pmol]* | **nominal**  **concentration**^1^  *[pmol/µL]* | **recovery^2^**  (mean ± sd, n = 4)  *[%]* | **matrix effect^3^ ESI(+)**  (mean ± sd, n = 4)  *[%]* | **matrix effect^3^ ESI(-)**  (mean ± sd, n = 4)  *[%]* |
| --- | --- | --- | --- | --- | --- | --- | --- | --- |
|  |  | *m/z* | *type* |  |  |  |  |  |
| 4-Aminohippuric acid | 1.60 | 120.0444 | [M+H-C_2_H_5_NO_2_]+ | 0.05 – 5 | 0.2 | 83.8 ± 2.8 | 37.5 ± 1.5 | 75.0 ± 5.3 |
|  |  | 193.0619 | [M-H]^-^ | 0.25 – 50 | 2 | 80.7 ± 8.0 | 31.5 ± 3.4 | 55.9 ± 4.5 |
| 3-Indoxyl β-D-glucoside | 4.10 | 134.0600 | [M+H-C_6_H_10_O_5_]^+^ | 0.5 – 25 | 2 | 90.6 ± 6.5 | 75.2 ± 2.1 | 81.6 ± 4.2 |
|  |  | 294.0983 | [M-H]^-^ | 1 - 50 | 20 | 96.9 ± 1.9 | 75.4 ± 3.2 | 79.1 ± 4.9 |
| Kinetin | 5.08 | 216.0880 | [M+H]^+^ | 0.025 – 5 | 0.05 | 93.4 ± 8.1 | 85.6 ± 5.0 | 70.5 ± 4.3 |
|  |  | 214.0734 | [M-H]^-^ | 0.025 – 5 | 0.5 | 98.0 ± 3.6 | 82.1 ± 3.3 | 64.6 ± 4.5 |
| Aspartame | 6.22 | 295.1289 | [M+H]^+^ | 0.025 – 5 | 0.1 | 91.2 ± 8.3 | 67.2 ± 3.6 | 74.7 ± 3.9 |
|  |  | 293.1143 | [M-H]^-^ | 0.1 – 25 | 1 | 95.5 ± 5.2 | 73.1 ± 4.7 | 68.0 ± 5.4 |
| Umbelliferone | 6.95 | 163.0390 | [M+H]^+^ | 0.05 – 2.5 | 0.05 | 93.8 ± 6.5 | 69.6 ± 3.5 | 86.4 ± 3.7 |
|  |  | 161.0244 | [M-H]^-^ | 0.025 – 5 | 0.5 | 99.8 ± 3.3 | 54.9 ± 3.1 | 77.7 ± 5.5 |
| Indole-3-carboxylic acid | 7.79 | 162.0550 | [M+H]^+^ | 0.25 – 10 | 0.5 | 96.7 ± 4.4 | 65.6 ± 2.6 | 113.7 ± 4.8 |
|  |  | 160.0404 | [M-H]^-^ | 0.5 – 50 | 5 | 98.0 ± 1.4 | 53.8 ± 1.8 | 91.3 ± 8.6 |
| Vitexin | 8.59 | 433.1129 | [M+H]^+^ | 0.05 – 5 | 0.1 | 95.2 ± 9.7 | 41.9 ± 2.5 | 77.9 ± 2.4 |
|  |  | 431.0984 | [M-H]^-^ | 0.025 – 5 | 1 | 98.8 ± 2.6 | 52.2 ± 2.6 | 79.4 ± 4.7 |
| Rutin | 9.45 | 611.1607 | [M+H]^+^ | 0.1 – 10 | 0.2 | 90.9 ± 6.4 | 75.0 ± 5.6 | 110.5 ± 8.1 |
|  |  | 609.1461 | [M-H]^-^ | 0.025 – 2.5 | 2 | 97.1 ± 3.1 | 53.8 ± 3.1 | 87.8 ± 4.3 |
| 4-Methoxycinnamic acid | 10.72/11.54 | 161.0597 | [M+H-H_2_O]^+^ | 0.25 – 25 | 1 | 97.8 ± 4.2 | 47.6 ± 4.1 | 62.8 ± 6.2 |
|  |  | 177.0557 | [M-H]^-^ | 1 – 100 | 10 | 95.8 ± 7.4 | 55.4 ± 2.8 | 74.0 ± 5.7 |
| Biochanin A | 15.58 | 285.0758 | [M+H]^+^ | 0.05 – 5 | 0.1 | 90.7 ± 7.3 | 14.1 ± 1.2 | 110.8 ± 5.5 |
|  |  | 283.0612 | [M-H]^-^ | 0.05 – 10 | 1 | 95.2 ± 5.4 | 11.1 ± 1.1 | 99.8 ± 6.9 |

^1^nominal concentration of the final extract, injection volume 1 µL

^2^recovery rate of Biochanin A was determined from data acquired in negative ion mode, for all other model compounds data from positive ion mode was used. ^3^matrix effect < 100%, ion suppression; matrix effect > 100%, ion enhancement

**Supplemental Table 3.** Inter-batch and intra-batch repeatabilities of 39 metabolites estimated from raw data or signal-drift corrected and normalized data.

| **no.** | **compound** | **t_r_**  *[min]* | **quantifier ion** | | **Coefficient of variation [%] calculated from raw data** | | | **Coefficient of variation [%] calculated from signal drift-corrected and normalized data** | | | |
| --- | --- | --- | --- | --- | --- | --- | --- | --- | --- | --- | --- |
|  |  |  | *m/z* | *type* | *inter-batch* | *intra-batch*  *extraction* | *intra-batch*  *instrumental* | *inter-batch* | *intra-batch*  *extraction* | *intra-batch*  *instrumental* | *total* |
| **N3** | 2´-Deoxyadenosine | 1.56 | 252.1091 | [M+H]^+^ | 25.4 | 1.7 | 2.2 | 2.4 | 2.0 | 1.9 | 3.5 |
| **N6** | 5´-Deoxy-5´-(methylthio)-adenosine | 3.92 | 298.0968 | [M+H]^+^ | 22.6 | 1.5 | 2.1 | 4.3 | 2.0 | 1.3 | 4.5 |
| **N11** | Thymidine | 2.37 | 127.0500 | [M+H-C_5_H_8_O_3_]^+^ | 29.1 | 0.3 | 4.7 | 1.8 | 1.0 | 4.0 | 4.4 |
| **N14** | Xanthosine | 2.03 | 153.0407 | [M+H-C_5_H_8_O_4_]^+^ | 28.4 | 0 | 3.2 | 2.1 | 1.4 | 2.4 | 3.3 |
| **A2** | Asp | 0.59 | 131.0462 | [M-H]^-^ | 30.9 | 0 | 14.6 | 1.2 | 0 | 4.4 | 4.5 |
| **A4** | pyro-Glu | 0.91 | 130.0499 | [M+H]^+^ | 27.2 | 0.8 | 3.9 | 4.8 | 1.4 | 3.1 | 5.5 |
| **A10** | Valine betaine | 0.92 | 160.1332 | [M+H]^+^ | 19.7 | 0.9 | 1.7 | 2.4 | 1.0 | 1.3 | 2.7 |
| **A18** | Hydroxypipecolic acid Hex | 2.89 | 308.1340 | [M+H]^+^ | 27.9 | 0 | 5.8 | 3.3 | 1.0 | 4.7 | 5.7 |
| **A27** | γ-Glu-Trp | 5.69 | 334.1398 | [M+H]^+^ | 30.6 | 0.6 | 4.1 | 2.9 | 1.5 | 2.8 | 4.1 |
| **A28** | Trp Hex | 2.60 | 367.1500 | [M+H]^+^ | 30.6 | 0 | 3.7 | 2.1 | 0.8 | 2.7 | 3.4 |
| **A29** | Kynurenic acid Hex | 1.91 | 190.0499 | [M+H-C_6_H_10_O_5_]^+^ | 31.0 | 0 | 4.6 | 3.0 | 1.0 | 3.2 | 4.3 |
| **A35** | Pyrosaccharopine | 1.91 | 259.1289 | [M+H]^+^ | 26.7 | 1.0 | 3.8 | 3.9 | 1.1 | 3.4 | 5.1 |
| **HQ2-1** | Methoxyhydroquinone *O*-Hex_3_ | 2.09 | 625.2985 | [M-H]^-^ | 28.4 | 0 | 13.9 | 1.8 | 0 | 2.7 | 3.1 |
| **HQ5-1&2** | 2,6-Dimethoxyhydroquinone *O*-Hex_2_ | 2.30/2.59 | 493.1563 | [M-H]^-^ | 48.3 | 0 | 21.0 | 5.0 | 0.8 | 3.8 | 6.0 |
| **HQ6-2** | 2,6-Dimethoxyhydroquinone *O*-(Hex-Sulfate) | 1.88 | 411.0603 | [M-H]^-^ | 50.1 | 0 | 19.3 | 7.1 | 3.5 | 3.6 | 8.1 |
| **HCAA-2** | *N*-Coumaroyl-hydroxyputrescine | 3.40 | 251.1390 | [M+H]^+^ | 27.8 | 6.8 | 3.8 | 11.5 | 6.4 | 3.8 | 12.7 |
| **HCAA11-1&2** | N^1^-Sinapoyl-agmatine | 4.78/6.26 | 337.1870 | [M+H]^+^ | 25.6 | 2.6 | 4.0 | 5.7 | 3.0 | 2.3 | 6.4 |
| **HCAA24-1&2** | N^x^-Coumaroyl-N^y^-(3,4-dimethoxycinnamoyl)-spermine | 5.38/6.02 | 539.3228 | [M+H]^+^ | 18.2 | 1.9 | 1.7 | 3.2 | 2.1 | 1.0 | 3.6 |
| **BX1** | 2-Hydroxy-1,4-benzoxazin-3-one 2-*O*-Hex_2_ | 4.83 | 488.1410 | [M-H]^-^ | 36.8 | 0 | 16.7 | 4.2 | 1.8 | 4.6 | 6.3 |
| **BX4** | 2,4-Dihydroxy-1,4-benzoxazin-3-one 2-*O*-Hex_2_ | 4.77 | 504.1359 | [M-H]^-^ | 26.8 | 0 | 13.2 | 2.8 | 0.9 | 2.7 | 3.8 |
| **P2** | Protocatechualdehyde | 3.67 | 137.0244 | [M-H]^-^ | 27.7 | 0 | 9.8 | 4.6 | 2.8 | 2.5 | 5.6 |
| **P7** | Protocatechuic acid | 2.77 | 153.0193 | [M-H]^-^ | 43.9 | 0 | 15.0 | 6.0 | 3.1 | 4.3 | 7.5 |
| **P12-1&2** | Ferulic acid isomer | 7.58/8.03 | 193.0506 | [M-H]^-^ | 37.8 | 0 | 11.0 | 3.3 | 3.3 | 3.8 | 5.8 |
| **P18** | Dehydrodiferulic acid | 10.07 | 385.0929 | [M-H]^-^ | 41.7 | 0 | 16.7 | 4.1 | 1.8 | 4.9 | 6.4 |
| **F2-3** | Apigenin *C*-Pent-*C*-Hex | 8.05 | 565.1552 | [M+H]^+^ | 20.7 | 0 | 2.6 | 1.0 | 0.5 | 1.2 | 1.5 |
| **F4** | Luteolin *C*-Pent-*C*-Hex | 7.34 | 581.1501 | [M+H]^+^ | 27.9 | 2.6 | 3.1 | 3.7 | 2.7 | 2.1 | 4.7 |
| **F5-2** | Chrysoeriol *C*-Pent-*C*-Hex | 8.50 | 595.1658 | [M+H]^+^ | 27.2 | 0 | 4.4 | 2.9 | 0.8 | 3.0 | 4.0 |
| **F9-1&2** | Apigenin *C*-Pent-*C*-Hex-*O*-Sinapoyl | 8.65/8.75 | 771.2131 | [M+H]^+^ | 18.2 | 0.6 | 2.9 | 2.1 | 1.4 | 1.4 | 2.8 |
| **F13** | Luteolin *O*-Hex-*O*-Hex | 8.15 | 609.1461 | [M-H]^-^ | 40.5 | 0 | 16.5 | 4.2 | 1.2 | 4.2 | 5.8 |
| **F16** | Tricin | 13.66 | 329.0667 | [M-H]^-^ | 28.8 | 0 | 13.7 | 4.9 | 5.7 | 3.8 | 8.1 |
| **F23** | Syringetin *O*-Hex-*O*-DeoxyHex | 10.90 | 653.1723 | [M-H]^-^ | 44.2 | 0 | 15.8 | 4.2 | 0 | 4.5 | 5.9 |
| **L1** | Hydroxymatairesinol | 10.35 | 373.1293 | [M-H]^-^ | 42.2 | 0 | 15.3 | 4.6 | 2.2 | 4.5 | 6.5 |
| **AS1** | Succinic acid | 1.23 | 117.0193 | [M-H]^-^ | 35.7 | 0 | 14.0 | 2.7 | 0 | 4.6 | 5.0 |
| **AS3** | Azelaic acid | 10.33 | 187.0976 | [M-H]^-^ | 39.2 | 0 | 7.3 | 4.1 | 1.3 | 3.1 | 5.0 |
| **AS6** | 2-Isopropylmalic acid | 4.77 | 175.0612 | [M-H]^-^ | 44.9 | 0 | 14.3 | 5.1 | 3.5 | 8.5 | 10.3 |
| **V1** | Pantothenic acid | 2.92 | 220.1180 | [M+H]^+^ | 31.1 | 0 | 5.4 | 3.3 | 0 | 3.9 | 4.8 |
| **V2** | Pantothenic acid *O*-Hex | 3.46 | 380.1562 | [M-H]^-^ | 24.0 | 0 | 12.5 | 3.2 | 0 | 3.2 | 4.3 |
| **V4** | Pyridoxin *O*-Hex | 0.87 | 332.1340 | [M+H]^+^ | 25.6 | 0.6 | 3.4 | 1.3 | 0 | 3.4 | 3.6 |
| **V10** | MeFox | 3.58 | 474.1732 | [M+H]^+^ | 21.9 | 1.0 | 2.9 | 1.2 | 0.9 | 2.6 | 3.0 |
| **Average** | | | | | **31.2** | **0.6** | **8.6** | **3.6** | **1.6** | **3.3** | **5.2** |

**Supplemental Table 4.** Nested linear random-effects model and its ANOVA table used for estimation of variance components within the repeatability experiment.

| $\boldsymbol{y}_{\boldsymbol{ijk}}\boldsymbol{=\mu+}\text{α}_{\boldsymbol{i}}\boldsymbol{+}\boldsymbol{\beta}_{\boldsymbol{ij}}\boldsymbol{+}\boldsymbol{\gamma}_{\boldsymbol{ijk}}$ | | | |
| --- | --- | --- | --- |
| $y_{ijk}$ | observed response of a given metabolite in batch i, extract j and injection k | | |
| $\mu$ | overall mean response | | |
| $\text{α}_{i}$ | independent random effect of batch i (i = 1, 2, 3, 4, 5), $\alpha_{i}\sim N\left( 0,\sigma_{batch}^{2} \right)$ | | |
| $\beta_{ij}$ | independent random effect of extract j in batch i (j = 1, 2, 3, 4, 5); $\beta_{ij}\sim N\left( 0,\sigma_{extr}^{2} \right)$ | | |
| $\gamma_{ijk}$ | independent random effect of injection k of extract j in batch i (k = 1, 2, 3, 4); $\gamma_{ijk}\sim N\left( 0,\sigma_{inj}^{2} \right)$ | | |
| **source of variation** | **degrees of freedom** | **sum of squares** | **expected value mean squares** |
| inter-batch | 5-1 | $5\times4\sum_{i=1}^{5} \left( \bar{y}_{i..}-\bar{y}_{\ldots} \right)^{2}$ | $\sigma_{inj}^{2}+4 \sigma_{extr}^{2}+5\times4\sigma_{batch}^{2}$ |
| intra-batch/extraction | 5 × (5-1) | $4\sum_{i=1}^{5} \sum_{j=1}^{5} \left( \bar{y}_{ij.}-\bar{y}_{i..} \right)^{2}$ | $\sigma_{inj}^{2}+4 \sigma_{extr}^{2}$ |
| intra-batch/instrument | 5 × 5 × (4-1) | $\sum_{i=1}^{5} \sum_{j=1}^{5} \sum_{k=1}^{4} {(y_{ijk}-\bar{y}_{ij.})}^{2}$ | $\sigma_{inj}^{2}$ |
| total | 5 × 5 × 4 - 1 | $\sum_{i=1}^{5} \sum_{j=1}^{5} \sum_{k=1}^{4} {(y_{ijk}-\bar{y}_{...})}^{2}$ | $\sigma_{tot}^{2}$ |

**Supplemental Table 5.** Evaluation of linearity by analysis of a serially diluted whole wheat grain extract. The coefficient of determination (R^2^) was determined within the greenish-labelled concentration range using a linear calibration model (peak area = a × concentration). X = chromatographic peak detected within the linear range, X = chromatographic peak detected outside the linear range due to saturation of the ion source or matrix effects, X = chromatographic peak detected outside the linear range due to saturation of the detector.

| **no.** | **compound** | **t_r_**  *[min]* | **quantifier ion** | | **extract concentration** *[µg dry weight/µL]* | | | | | | | | | | | **R^2^** |
| --- | --- | --- | --- | --- | --- | --- | --- | --- | --- | --- | --- | --- | --- | --- | --- | --- |
|  |  |  | *m/z* | *type* | 4 | 8 | 20 | 30 | 37.5 | 50 | 75 | 100 | **150*** | 300 | 400 |  |
| **N3** | 2´-Deoxyadenosine | 1.56 | 252.1091 | [M+H]^+^ | X | X | X | X | X | X | X | X | X | X | X | 0.989 |
| **N6** | 5´-Deoxy-5´-(methylthio)-adenosine | 3.92 | 298.0968 | [M+H]^+^ | X | X | X | X | X | X | X | X | X | X | X | 0.998 |
| **N11** | Thymidine | 2.37 | 127.0500 | [M+H-C_5_H_8_O_3_]^+^ | X | X | X | X | X | X | X | X | X | X | X | 0.954 |
| **N14** | Xanthosine | 2.03 | 153.0407 | [M+H-C_5_H_8_O_4_]^+^ | X | X | X | X | X | X | X | X | X | X | X | 0.996 |
| **A2** | Asp | 0.59 | 131.0462 | [M-H]^-^ | X | X | X | X | X | X | X | X | X | X | X | 0.856 |
| **A4** | pyro-Glu | 0.91 | 130.0499 | [M+H]^+^ | X | X | X | X | X | X | X | X | X | X | X | 0.881 |
| **A10** | Valine betaine | 0.92 | 160.1332 | [M+H]^+^ | X | X | X | X | X | X | X | X | X | X | X | 0.988 |
| **A18** | Hydroxypipecolic acid Hex | 2.89 | 308.1340 | [M+H]^+^ | X | X | X | X | X | X | X | X | X | X | X | 0.996 |
| **A27** | γ-Glu-Trp | 5.69 | 334.1398 | [M+H]^+^ | X | X | X | X | X | X | X | X | X | X | X | 0.995 |
| **A28** | Trp Hex | 2.60 | 367.1500 | [M+H]^+^ | X | X | X | X | X | X | X | X | X | X | X | 0.964 |
| **A29** | Kynurenic acid Hex | 1.91 | 190.0499 | [M+H-C_6_H_10_O_5_]^+^ | X | X | X | X | X | X | X | X | X | X | X | 0.985 |
| **A35** | Pyrosaccharopine | 1.91 | 259.1289 | [M+H]^+^ | X | X | X | X | X | X | X | X | X | X | X | 0.999 |
| **HQ2-1** | Methoxyhydroquinone *O*-Hex_3_ isomer #1 | 2.09 | 625.2985 | [M-H]^-^ | X | X | X | X | X | X | X | X | X | X | X | 0.943 |
| **HQ5-1** | 2,6-Dimethoxyhydroquinone *O*-Hex_2_ isomer#1 | 2.30 | 493.1563 | [M-H]^-^ | X | X | X | X | X | X | X | X | X | X | X | 0.990 |
| **HQ6** | 2,6-Dimethoxyhydroquinone *O*-(Hex-Sulfate) | 1.88 | 411.0603 | [M-H]^-^ | X | X | X | X | X | X | X | X | X | X | X | 0.989 |
| **HCAA2-2** | *N*-Coumaroyl-hydroxyputrescine isomer #2 | 3.40 | 251.1390 | [M+H]^+^ | X | X | X | X | X | X | X | X | X | X | X | 0.997 |
| **HCAA11-2** | *N*^1^-Sinapoyl-agmatine isomer #2 | 6.26 | 337.1870 | [M+H]^+^ | X | X | X | X | X | X | X | X | X | X | X | 0.993 |
| **HCAA24-2** | *N*^x^-Coumaroyl-*N*^y^-(3,4-dimethoxycinnamoyl)-spermine isomer #2 | 6.02 | 539.3228 | [M+H]^+^ | X | X | X | X | X | X | X | X | X | X | X | 0.998 |
| **BX1** | 2-Hydroxy-1,4-benzoxazin-3-one 2-*O*-Hex_2_ | 4.83 | 488.1410 | [M-H]^-^ | X | X | X | X | X | X | X | X | X | X | X | 0.995 |
| **BX4** | 2,4-Dihydroxy-1,4-benzoxazin-3-one 2-*O*-Hex_2_ | 4.77 | 504.1359 | [M-H]^-^ | X | X | X | X | X | X | X | X | X | X | X | 0.964 |
| **P2** | Protocatechualdehyde | 3.67 | 137.0244 | [M-H]^-^ | X | X | X | X | X | X | X | X | X | X | X | 0.990 |
| **P7** | Protocatechuic acid | 2.77 | 153.0193 | [M-H]^-^ | X | X | X | X | X | X | X | X | X | X | X | 0.940 |
| **P12-2** | Ferulic acid isomer #2 | 8.03 | 193.0506 | [M-H]^-^ | X | X | X | X | X | X | X | X | X | X | X | 0.953 |
| **P18** | Dehydrodiferulic acid | 10.07 | 385.0929 | [M-H]^-^ | X | X | X | X | X | X | X | X | X | X | X | 0.993 |
| **F2-3** | Apigenin *C*-Pent-*C*-Hex isomer #3 | 8.05 | 565.1552 | [M+H]^+^ | X | X | X | X | X | X | X | X | X | X | X | 0.931 |
| **F4** | Luteolin *C*-Pent-*C*-Hex | 7.34 | 581.1501 | [M+H]^+^ | X | X | X | X | X | X | X | X | X | X | X | 0.996 |
| **F5-2** | Chrysoeriol *C*-Pent-*C*-Hex isomer #2 | 8.50 | 595.1658 | [M+H]^+^ | X | X | X | X | X | X | X | X | X | X | X | 0.997 |
| **F9-1&2** | Apigenin *C*-Pent-*C*-Hex-*O*-Sinapoyl isomer #1 & #2 | 8.65/8.75 | 771.2131 | [M+H]^+^ | X | X | X | X | X | X | X | X | X | X | X | 0.989 |
| **F13** | Luteolin *O*-Hex-*O*-Hex | 8.15 | 609.1461 | [M-H]^-^ | X | X | X | X | X | X | X | X | X | X | X | 0.948 |
| **F16** | Tricin | 13.66 | 329.0667 | [M-H]^-^ | X | X | X | X | X | X | X | X | X | X | X | 0.993 |
| **F23** | Syringetin *O*-Hex-*O*-DeoxyHex | 10.90 | 653.1723 | [M-H]^-^ | X | X | X | X | X | X | X | X | X | X | X | 0.991 |
| **L1** | Hydroxymatairesinol | 10.35 | 373.1293 | [M-H]^-^ | X | X | X | X | X | X | X | X | X | X | X | 0.994 |
| **AS1** | Succinic acid | 1.23 | 117.0193 | [M-H]^-^ | X | X | X | X | X | X | X | X | X | X | X | 0.772 |
| **AS3** | Azelaic acid | 10.33 | 187.0976 | [M-H]^-^ | X | X | X | X | X | X | X | X | X | X | X | 0.970 |
| **AS6** | 2-Isopropylmalic acid | 4.77 | 175.0612 | [M-H]^-^ | X | X | X | X | X | X | X | X | X | X | X | 0.999 |
| **V1** | Pantothenic acid | 2.92 | 220.1180 | [M+H]^+^ | X | X | X | X | X | X | X | X | X | X | X | 0.975 |
| **V2** | Pantothenic acid *O*-Hex | 3.46 | 380.1562 | [M-H]^-^ | X | X | X | X | X | X | X | X | X | X | X | 0.986 |
| **V4** | Pyridoxin *O*-Hex | 0.87 | 332.1340 | [M+H]^+^ | X | X | X | X | X | X | X | X | X | X | X | 0.939 |
| **V10** | MeFox | 3.58 | 474.1732 | [M+H]^+^ | X | X | X | X | X | X | X | X | X | X | X | 0.982 |

*working concentration for metabolite profiling

**Supplemental Table 6.** Accuracy of relative quantification determined for six differentially spiked test compounds.

| **Test compound**  **(lowest fortification level)** |  | **Nominal FC^1^** | | | | | | | | |
| --- | --- | --- | --- | --- | --- | --- | --- | --- | --- | --- |
|  |  | **1.05** | **1.10** | **1.15** | **1.20** | **1.25** | **1.50** | **2.00** | **4.00** | **8.00** |
| 4-Aminohippuric acid  (0.5 µM) | exp FC^2^ | 1.07 ± 0.01 | 1.11 ± 0.03 | 1.18 ± 0.04 | 1.23 ± 0.02 | 1.32 ± 0.01 | 1.64 ± 0.02 | 2.28 ± 0.09 | 4.99 ± 0.20 | 10.49 ± 0.39 |
|  | -log_10_ *P*^3^ | 3.80 | 2.41 | 2.76 | 4.72 | 6.22 | 6.40 | 4.74 | 5.47 | 5.77 |
| 3-Indoxyl β-D-glucoside  (2 µM) | found FC^2^ | 1.08 ± 0.02 | 1.15 ± 0.03 | 1.21 ± 0.04 | 1.30 ± 0.02 | 1.37 ± 0.03 | 1.73 ± 0.06 | 2.42 ± 0.06 | 5.08 ± 0.33 | 10.00 ± 1.25 |
|  | -log_10_ *P*^3^ | 2.84 | 3.04 | 3.19 | 4.97 | 5.25 | 4.75 | 6.12 | 4.62 | 3.64 |
| Kinetin  (0.1 µM) | found FC^2^ | 1.07 ± 0.04 | 1.15 ± 0.04 | 1.21 ± 0.04 | 1.28 ± 0.05 | 1.37 ± 0.04 | 1.71 ± 0.06 | 2.44 ± 0.07 | 4.96 ± 0.17 | 10.12 ± 0.39 |
|  | -log_10_ *P*^3^ | 1.42 | 2.81 | 3.48 | 3.27 | 4.20 | 4.66 | 6.19 | 6.25 | 6.13 |
| Aspartame  (0.2 µM) | found FC^2^ | 1.07 ± 0.01 | 1.16 ± 0.03 | 1.21 ± 0.03 | 1.30 ± 0.04 | 1.38 ± 0.01 | 1.74 ± 0.04 | 2.55 ± 0.06 | 5.83 ± 0.19 | 12.22 ± 0.74 |
|  | -log_10_ *P*^3^ | 4.47 | 3.23 | 3.35 | 3.89 | 6.32 | 5.17 | 5.72 | 5.83 | 4.91 |
| Umbelliferone  (0.2 µM) | found FC^2^ | 1.07 ± 0.02 | 1.15 ± 0.03 | 1.19 ± 0.02 | 1.25 ± 0.03 | 1.34 ± 0.03 | 1.64 ± 0.05 | 2.29 ± 0.05 | 4.64 ± 0.15 | 8.87 ± 1.26 |
|  | -log_10_ *P*^3^ | 2.56 | 2.91 | 3.83 | 4.21 | 4.58 | 4.87 | 6.07 | 5.82 | 3.40 |
| Biochanin A  (0.5 µM) | found FC^2^ | 1.03 ± 0.09 | 1.15 ± 0.08 | 1.21 ± 0.07 | 1.17 ± 0.06 | 1.30 ± 0.11 | 1.51 ± 0.09 | 2.48 ± 0.35 | 5.13 ± 0.73 | 10.01 ± 0.56 |
|  | -log_10_ *P*^3^ | 0.21 | 1.66 | 1.44 | 2.49 | 2.08 | 3.52 | 2.80 | 3.29 | 5.83 |

^1^ nominal (theoretical) fold change of the spiked test compound between two concentration levels

^2^ experimentally determined fold change of the spiked test compound between two concentration levels, [mean concentration level 1 (n = 3) / mean concentration level 2 (n = 3)] ± sd is shown

^3^ Student´s *t*-test (two-tailed) was used to evaluate if the means of two concentration levels are significantly different (df = 4)
